# Measuring accessibility to public services and infrastructure criticality for disasters risk management

**DOI:** 10.1038/s41598-023-28460-z

**Published:** 2023-01-28

**Authors:** Mersedeh Tariverdi, Miguel Nunez-del-Prado, Nadezda Leonova, Jun Rentschler

**Affiliations:** The World Bank, Washington, USA

**Keywords:** Health care, Public health

## Abstract

Component criticality analysis of infrastructure systems has traditionally focused on physical networks rather than infrastructure services. As an example, a key objective of transport infrastructure is to ensure mobility and resilient access to public services, including for the population, service providers, and associated supply chains. We introduce a new user-centric measure for estimating infrastructure criticality and urban accessibility to critical public services - particularly healthcare facilities without loss of generality - and the effects of disaster-induced infrastructure disruptions. Accessibility measures include individuals’ choices of all services in each sector. The approach is scalable and modular while preserving detailed features necessary for local planning decisions. It relies on open data to simulate various disaster scenarios, including floods, seismic, and compound shocks. We present results for Lima, Peru, and Manila, Philippines, to illustrate how the approach identifies the most affected areas by shocks, underserved populations, and changes in accessibility and critical infrastructure components. We capture the changes in people’s choices of health service providers under each scenario. For Lima, we show that the floods of 2020 caused an increase in average access times to all health services from 33 minutes to 48 minutes. We identify specific critical road segments for ensuring access under each scenario. For Manila, we locate the 22% of the population who lost complete access to all higher health services due to flooding of over 15 cm. The approach is used to identify and prioritize targeted measures to strengthen the resilience of critical public services and their supporting infrastructure systems, while putting the population at the center of decision-making.

## Introduction

Ensuring efficient access to critical public services, such as healthcare and education, is already a challenging imperative for policymakers in regular times. However, during crises, such as climatic and natural shocks, these services’ needs and operating environments become even more complex. Evidence-based assessments of the hazards, exposure, and vulnerabilities are crucial for taking targeted measures to strengthen people’s resilience and the public services and infrastructure on which they depend during crises.

Different studies have been performed to assess social vulnerability^1–3^, accessibility^4–7^, criticality^8–11^, infrastructure systems interdependence^12,13^, and optimal placement of public service facilities^14^. These studies provide evidence to policymakers to protect access to essential public services like healthcare in normal conditions and during shocks such as floods. To this end, they need to understand how the disruptions would affect the population’s access to services, either direct access to physical infrastructure and/or disruption to telecommunication services which would affect communication efforts to organize rescue services. On this note, infrastructure resiliency investments must reflect the importance of availability and access to public services, as in emergency preparedness and mitigation interventions. Therefore, we suggest using population accessibility and infrastructure criticality metrics based on intended functionality to quantify, manage, and guarantee access to essential public services, and improve those services’ resiliency through cross-sectoral investments. For demonstration, healthcare is selected for public service and transportation network as the supporting infrastructure. The following paragraphs present examples of prior works on accessibility, network criticality, and resiliency approaches and methods.

Recently, accessibility has been studied widely across different domains, namely transportation^15^, governmental services^16^, parks access^17^, jobs access^18^, health access^19^, and more recently access to COVID-19 services^20^. Accessibility metrics are crucial to understanding urban dynamics. The work of Levinson and Wu^21^ generalizes the concept of accessibility and details different aspects about origin locations of trips; the impedance function in terms of time, distance, and economical cost; how to assess primary access, when the travel begins; the travel purpose; the transportation mode; and the access for different population groups. The authors focus on all the elements intervening in the accessibility and methodology applied to all public services. Similarly, relevant to services, Jiao *et al.*^22^ study accessibility to the food market for detecting food deserts. The authors find that food deserts are dependent on the definition and measurement of incomes, as well as economic and physical access to supermarkets. Regarding accessibility to jobs, Barboza *et al.*^18^ propose a new metric for assessing accessibility named *balancing time*, which represents the minimum time spent in public transportation to find job opportunities for the working-age population of a given area.

Accessibility to health services has also been comprehensively studied in the literature. Wang *et al.*^23^ propose an extension of the Two-step Floating Catchment Area Method (2SFCA) model^24^ to estimate supply and demand. They consider population data in a $1\times 1$-Km grid form, general and specialized healthcare facilities, number of beds per healthcare facility, taxi trips, road network from OpenStreetMaps (OSM), and travel time estimates as inputs. The authors find a traffic jam congestion factor of 2.89, which almost triples travel times relative to ideal conditions. Using the same model, Kiani *et al.*^25^ analyse accessibility in terms of travel time to the closest hospitals. The authors used neighbourhood centroids as origins from which to compute the closest distance to hospitals. In another study, Zhao, Li, and Liu^26^ examine the role of public transport networks of buses and subways in public healthcare facilities’ accessibility using E2SFCA. Consequently, to compute travel times from neighbourhoods’ centroids to healthcare facilities, the Baidu Map API was used. Based on this information, they found that suburban neighbourhoods have more restricted access to health services than neighbourhoods located in the central urban area.

Qian *et al.*^27^ study the accessibility differences of Nanjing, China, hospitals grouped by patients reaching the hospitals by public or private transportation. The authors use the Gaussian 2SFCA model to compute accessibility from $1\times 1$Km grid centroids to hospitals, also relying on travel times from Baidu Maps API. They find that private transportation is preferred for accessing hospitals due to time differences ranging from 12-19 minutes in private transport to 40-59 minutes in public transportation (subway and buses). In the same spirit, Boisjoly *et al.*^28^ analyze the healthcare accessibility of eight different cities in Canada using the 2SFCA model with a temporal threshold of 45 minutes. The authors focused their study on accessibility when using only public transport, and identify vulnerable census tracts with limited healthcare access. Zhou *et al.*^29^ propose a new model to quantify accessibility based on multi-modal (public transportation, driving, walking, and bicycling) transportation combined with the residential transportation mode choices over a 2SFCA model. The authors use an empirical origin-destination matrix, detailing trips from respective street blocks to two pediatric hospitals. They show that their model could more accurately measure accessibility compared to a single-mode transportation model. Tao *et al.*^30^ use the GV2SFCA model to measure the fastest travel time of private vehicles as an accessibility indicator to hospitals at the town level. Alabbad *et al.*^20^ measures accessibility to the closest critical amenity using the Dijkstra shortest-path algorithm at the node level provided by OSM. Finally, Kang *et al.*^20^ study the hospitals’ accessibility in Illinois to assess access to health services during the COVID-19 pandemic. They use a parallel version of the E2SFCA model based on the OSM road network with time thresholds of 10, 20, and 30 minutes. The authors focused their study on hospitals with Intensive Care services using the Dijkstra algorithm to find the shortest path from hexagon grid centroids to the closest hospital. They identified geographic areas needing additional healthcare resources to improve access. Table S1 summarises the assumptions of the before-described studies.

Beyond identifying the vulnerable areas and populations for service planning, accessibility can also be used in the context of infrastructure resiliency analysis and the criticality of roadways, as proposed in this work. While the former metric measures how difficult access through a road network is, the latter quantifies how important a road segment, or any network component, is in the whole network to guarantee access in normal and disrupted times. Infrastructure criticality has been studied widely in the literature for lifelines such as power and transport. For instance, Kim and Yeo^31^ use Macroscopic Fundamental Diagram (MDF) to evaluate the link criticality of road networks. The MDF is a diagram that relates the space flow density of a road network. The authors use the MDF to evaluate congestion and road disruption due to an extreme event in the Gangnam district of Seoul, Korea. The authors showed that flow-based techniques could be equivalent to time-based methods for criticality measures. Colon *et al.*^32^ analyze Tanzania’s road network criticality coupled with flood hazard data and a production and demand network of food supplies. Therefore, the authors study the economic impact of link disruption vis-a-vis the country’s activities and food security. Another study by Garcia-Palomares *et al.*^33^ proposes a framework to measure the criticality of the Spanish road network. The authors consider data analysis based on accessibility metrics, such as travel time, potential accessibility, and daily accessibility. Similarly, Su *et al.*^34^ quantify the road criticality of a section of Beijing’s road network by relying on the temporal and spatial features of traffic flow. Thus, the criticality in a section is measured by traffic flow contribution to the network and influence on other sections simultaneously.

With few exceptions, most research focused on analyzing access to amenities and the criticality of individual road infrastructure elements. Regarding accessibility, most studies are based on the 2SFCA-based method and the closest amenity. The former computes the travel distance based on the shortest time, without taking into account congestion in the catchment area^35^ of the amenity under study^20,23–25,27–30^. The latter relies on the shortest path to the closest healthcare facilities^20,26^. It is important to highlight that traditionally accessibility has been measured from the provider’s perspective. Concerning criticality of the road network, the works in the literature consider a single perturbation in the network due to external events^31,36^, floods^32^, disruption^33^ or congestion^34^. Many studies rely on methods that require very specific data that are not commonly available, especially in low- and middle-income countries. In addition, the analyzed studies estimate population accessibility and infrastructure criticality at a relatively coarse resolution (i.e., neighbourhoods, or municipalities) to manage the computational burden. Some works also analyze compound shocks that overlap in space and time^37–39^. In this work, we propose a new methodology based on a whole-of-system approach^40^. The aim of our methodology is to assess population accessibility and infrastructure criticality. Accessibility measures population access to public services from the user perspective. Road infrastructure criticality, on the other hand, measures how crucial the road segments are with regard to the population and services they support. Our approach relies on readily available open data for rapid assessments and reduces computational costs relative to existing approaches while preserving a high level of granularity to support decision-making in data-scarce settings.

More precisely, our contributions are the following:

1. *Accessibility* We propose a new accessibility metric that takes into account people’s choices to represent expected travel times to healthcare services (without losing generality to other sectoral services). Thus, the present metric goes beyond classical approaches of measuring travel time within a catchment area of a single facility^20,23–25,27–30^ or to the closest facility^20,26^. This metric captures people’s preference for services in terms of treatment type, distance, and healthcare facility capacity and aggregates access to all services.
2. *Criticality*: We propose a new metric to quantify the importance of road segments at the street level based on accessibility measures introduced and weighted by population and service type. Our metric allows us to capture the direct and indirect impact of multiple natural hazards on access, which overlaps spatially and, in some cases, temporally.
3. *Isolation* This metric quantifies the population at risk of being isolated from the systems under study due to the disruptive impacts of natural hazards on road infrastructure.
4. *Population’s service preference* Our model captures individuals’ preferences and needs by computing the probability of going to all healthcare facilities based not only on the distance to the facility but also on the complexity, and capacity of the facility. Current studies in the literature do not capture this users’ perspective decision process in accessibility analysis. Additionally, our method quantifies the changes in probabilities of users’ choices in the presence of hazards relative to the baseline scenario without disruptions.

The next section explains the details of the methodology, which is based on the topology of the infrastructure network, the location of public services carrying the relevant information on the service, and population distribution. The analytical challenge consists in ensuring the computational feasibility, while preserving sufficient granularity to allow urban planners to readily use results (*e.g.*, to identify critical road segments with respect to accessibility to health services with and without hazard-induced disruptions). Our methodology allows us to study healthcare as a singular connected system and evaluate its interaction with other public services, thus enabling a cross-sectoral *health in all policies* approach^41^. While this study focuses on the healthcare system, its metrics and frameworks are directly applicable to other public services and sectors.

## Methods

**Figure 1:**
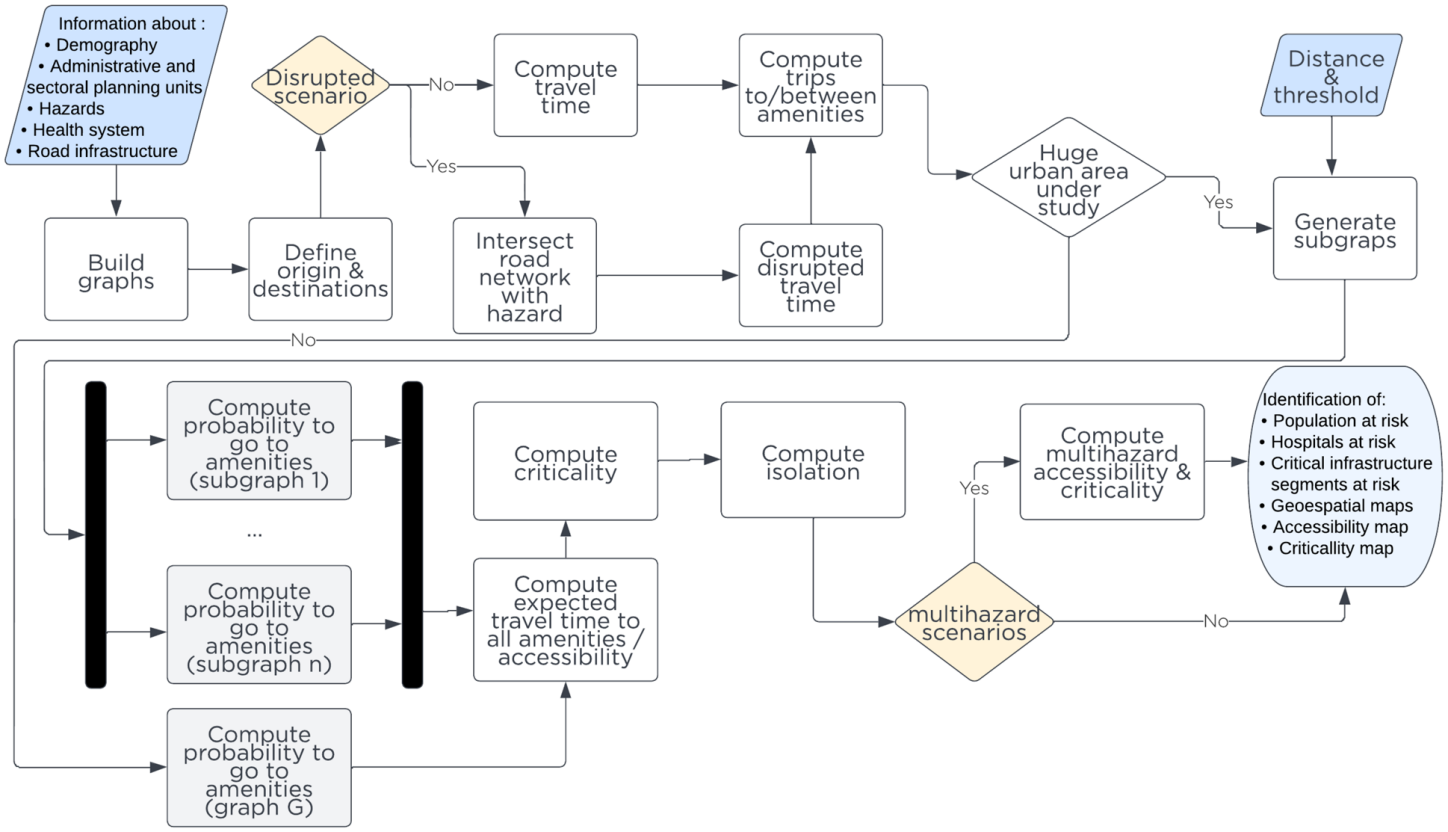
Methodology schema of population accessibility and infrastructure criticality estimation.

This section proposes a new methodology for computing people’s access to the essential public services of their choice, and estimating infrastructure criticality while accounting for the services they provide to users. This method relies on open data and reduces computational costs without losing the granularity needed for practical planning and investment decisions. Here, *accessibility* quantifies the average expected time to access all public services in a sector, and *criticality* measures how crucial a road segment is in ensuring access for the population. The following paragraphs describe the method to compute accessibility and criticality metrics for infrastructures, as depicted in Fig. 1. We present examples from the healthcare system to explain our proposal, though its relevance extends to other sectors and public services.

General categories of inputs for this model are the boundaries of the study area, including administrative and service planning (i.e. health sector planning divisions), population density, hazard maps, the road network, and the average travel speeds on different road types. The baseline graph *G*(*V*, *E*) represents the road network within the study area, where the vertex *V* are street intersections, and edges *E* represent streets. The tool to extract the road network is OSMnx library^42^ from the OpenStreetMap (OSM) service^43^. Once the road network *G* is built, the important amenities representing the public service locations, like *healthcare facilities*, are snapped to the vertices *V* using the GOSTNets Library^44^ as depicted in Fig. S1.

**Figure 2:**
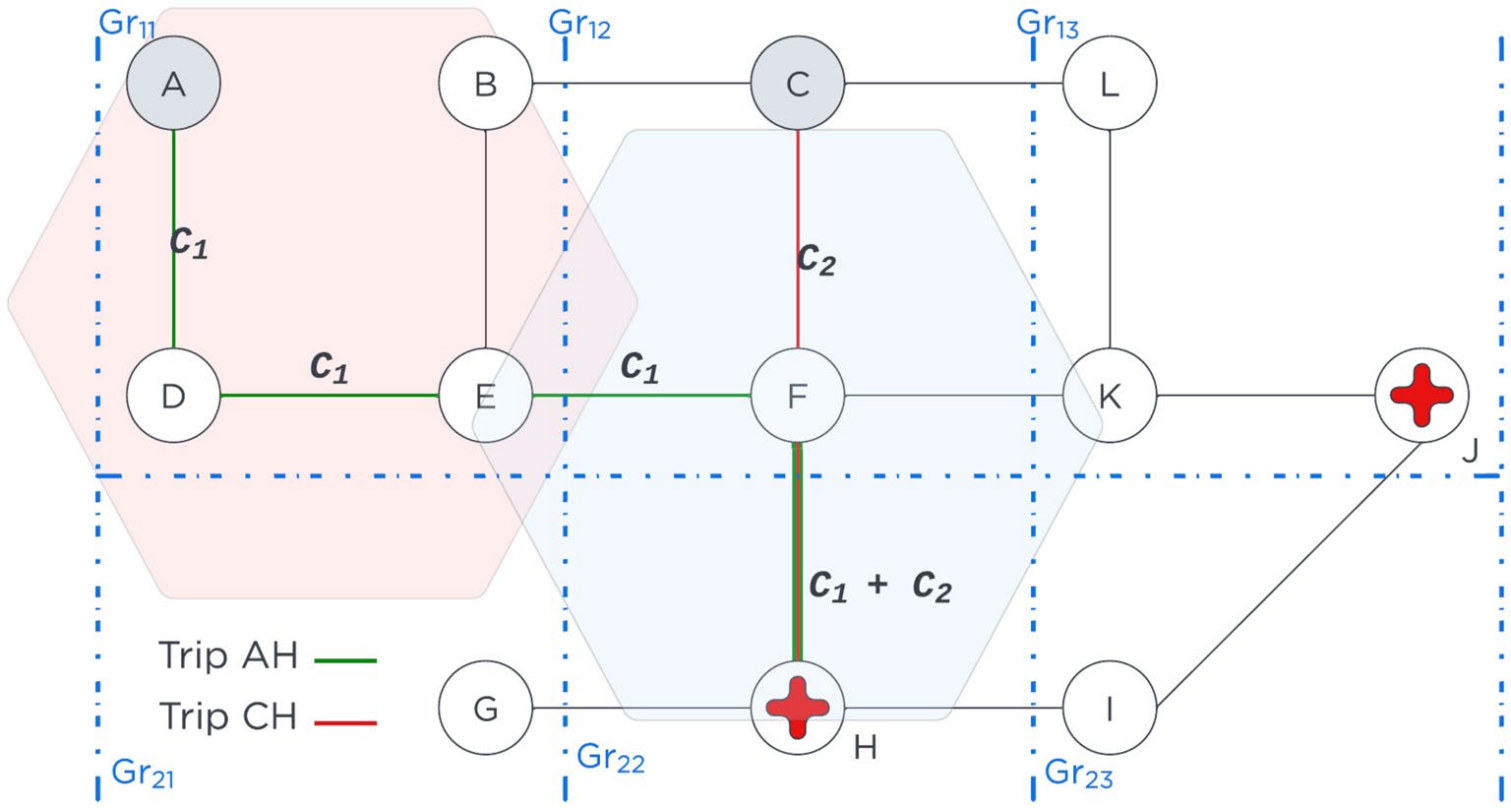
Simplified hypothetical city example for methodology illustration purposes.

The *origin and destination* points are selected on the road network infrastructure represented by graph *G*. For the origin points, five strategies are proposed to create a subsample $S_{sample}$ of the origin vertex to represent the population distribution and density. The first strategy is to use a fixed grid of $1Km \times 1Km$. The second relies on the centroids of a population density grid $P_{grid}$. The third takes 10% of the closest nodes to the centroid node within each polygon defined by the population density grid; The fourth and fifth strategies take 10% or 5% of random points within a population density grid. Service facilities locations in latitude and longitude are snapped to the baseline network and considered as the destination points. For example, Fig. 2 illustrates the origin points as grey nodes and destinations as nodes with a red cross. Additionally, the population of the grid is proportionally distributed to each selected node as an origin. In the example of Fig. 2, if nodes *A*, *B*,  and *D* were chosen as origins, they each are assigned one-third of the population of the grid $Gr_{11}$.

At the *hazard intersection* step, we verify whether a road segment $\overline{\textrm{XY}} \in E$ is affected by one or more hazard events $\phi$ (i.e. earthquakes, floods, etc.), which could overlap spatially and temporally. The impact of a single or compound natural shocks over the affected road segments is captured by reduction of the speed attribute $\overline{\textrm{XY}}_{speed}$ as shown in Eq. 1 or total link disruption.

1 $$\begin{aligned} \overline{\textrm{XY}}_{speed}= \left\{ \begin{array}{ll} \frac{\overline{\textrm{XY}}_{speed}}{\rho } , &{} {\text {if } \overline{\textrm{XY}} \cap \phi \ne \emptyset } \\ \overline{\textrm{XY}}_{speed}, &{} otherwise \end{array} \right. \end{aligned}$$

Where $\rho$ is a speed penalization factor. For the sake of clarity, in Fig. 2, there are two hexagons, red $\phi _{\text {fault lines }}$ and blue $\phi _{\text {floods }}$ representing the impact of the natural hazards over graph *G*. Consequently, $\overline{\textrm{AD}}'_{speed}, \overline{\textrm{DE}}'_{speed}, \overline{\textrm{EB}}'_{speed}, \overline{\textrm{EF}}'_{speed} \in E$ road segments are affected by the fault lines $\phi _{\text {fault lines}}$, while $\overline{\textrm{EF}}'_{speed}, \overline{\textrm{CF}}'_{speed}, \overline{\textrm{FK}}'_{speed}, \overline{\textrm{FH}}'_{speed}, \overline{\textrm{GH}}'_{speed}, \overline{\textrm{HI}}'_{speed} \in E$ are concerned by the floods $\phi _{\text {floods}}$. This implies that speed in a given road segment $\overline{\textrm{XY}}_{speed}$ is higher in the absence of hazards $\overline{\textrm{XY}}_{speed} > \overline{\textrm{XY}}'_{speed}$. In addition, the $\overline{\textrm{EF}}$ segment is affected by fault lines and floods. Note that speed loss is a proxy for road network infrastructure utility loss in the absence of accurate infrastructure exposure and fragility information. Where available, ground-truthed empirical information could complement the estimated vulnerability of public services and infrastructure. This approach allows performing multi-risk assessments of different natural hazards, which could overlap spatially and, in some cases, temporally depending on the risk profile of countries. Examples of compound shocks include earthquake, wind, and flood hazards in the Philippines^37^; heat waves, inland flooding, and coastal flooding in New York City, USA^38^; combined seismic and flood risks in Emilia-Romagna, Italy^45^; flood inundation and landslides in Golestan, Iran^39^; and landslides caused by monsoons in the Himalayan Nation and rainfall-triggered landslides due to earthquake in 2015 in Gorkha, Nepal^46^.

Following this framework, travel times are computed for baseline and disrupted scenarios. Computing the probability of reaching amenities is more computationally intensive in larger and denser areas as the complete origin-destination matrix increases. Therefore the computational complexity of this operation is $O(n \times m)$, where *n* are the number of origins and *m* the number of destinations. When the analyzed urban area is large, we divide the graph *G* into subgraphs $G_i$ based on an influence radius $\lambda$ in Km from amenities’ (i.e., healthcare facility) location to improve the computation time and reduce the complexity in such settings. Subgraphs $G_i$ sharing at least $\delta$ percent of nodes are merged to address duplication. This allows parallel processing of subgraphs $G_i$. Estimated travel time $t_{S_i,j}$ of users between subgraphs, for amenities located outside of the subgraph, from one origin in subgraph $G_i$ to another amenity in subgraph $G_j$ is the shortest path from the origin point to the amenity in subgraph $G_i$ plus the travel time from amenities in graphs $G_i$ and $G_j$, respectively. For example, in Fig.2 consider that nodes in the grids $Gr_{11}, Gr_{12}, Gr_{21}$, and $Gr_{22}$ are one subgraph. Thus, to compute the travel time from *A* to *J* $t_{S_A,J}$, we take the travel time from *A* to *H* $t_{S_A,H}$ plus the travel time from *H* to *J* $t_{S_H,J}$ $t_{S_A,J} = t_{S_A,H} + t_{S_H,J}$. To compute travel time, we rely on the Dijkstra algorithm weighted by the speed of the edges. Another consideration to highlight is the use of driving travel time due to individuals’ preference to reach healthcare facilities^27^. In addition, no particular time window for a trip to start is considered. Thus, while we do not account for traffic levels, the speed feature could account for a congestion factor if such information were available^47^. The estimated travel times from this analysis are representative of off-peak hours.

In this present approach, travel times and healthcare capacity are used in Huff’s attractivity function. Using the example of the health sector, we generalize the attractivity function based on the population accessibility to services, capacity, and quality of services given the availability of information. This is meant to capture Users’ choices in prioritizing where to seek services. For the health sector in this work, we use time travel and capacity as proxies in absence of validated data on all facilities. The area of a healthcare facility is used as the proxy to the health facility category in providing a complex set of services and capacity. Larger facilities are more likely to be higher care services with more patient throughput for example. Nonetheless, if the number of beds, physicians, nurses, *etc*. are available, they could be integrated into the Attractivity function of the Huff model. However, not all countries possess or make such information publicly available. Once subsampled origins and destinations are fixed, the probabilities are calculated in parallel from sampled origins $S_i$ to destinations $j \in J$. Using the time $t_{S_i,j}$ to reach the amenities from $S_i$ and the amenities’ area $A_j$ as input to the Huff model^48^, the probability $P_{s,j}$ to visit all amenities (i.e., healthcare facilities) is computed using Eq. 2.

2 $$\begin{aligned} P_{S_i,j}=\frac{\frac{A_j}{t_{S_i,j}}}{ \sum _{S_i}^{S}\frac{A_j}{t_{S_i,j}} } \end{aligned}$$

Therefore, taking Fig. 2, at the end of this stage, the probabilities $P_{AH}, P_{CH}, P_{AJ},$ and $P_{CJ}$ to reach healthcare facilities *H* and *J* from nodes *A* and *C* are computed; where $P_{AH} + P_{AJ} = 1$ and $P_{CH} + P_{CJ} = 1$. Note that probabilities from one node to the amenities like $P_{AH}$ and $P_{AJ}$ also characterize the facility preference, which could change in the presence of hazards.

Having the complete O-D matrix, road infrastructure metrics like accessibility, criticality, and isolation are computed. *Accessibility* measures the average time for individuals to reach the service system under study and not only the time to the closest facility. *Criticality* quantifies the importance of each segments in the road network infrastructure that enables people to reach the service system under study, such as healthcare or education. Finally, *isolation* represents the share of the population experiencing disrupted road network infrastructure, which stops them from accessing service systems.

Accessibility represents the expected travel time to access the service system under study. Accessibility is the sum of the time from a node $S_i$ to reach all the amenities *j* weighted by the probability $P_{S_i,j}$ to reach this amenity, as shown in Eq. 3.

3 $$\begin{aligned} Accessibility_{S_i} = \sum _{j \in J}^{|J|} t_{S_i,j} \cdot P_{S_i,j} \end{aligned}$$

For instance, if travel times and probabilities from node A to reach healthcare facilitys *H* and *J* are denoted by $t_{AH}$, $t_{AJ}$, $P_{AH}$, and $P_{AJ}$, respectively in Fig.2, then accessibility time for node *A* will be $t_{AH} \times P_{AH} + t_{AJ} \times P_{AJ}$. The criticality of the road segments regarding a single service is built based on population density and preference-weighted edge betweenness centrality. To compute criticality, we consider the population at origin $w_{S_i}$ weighted by the probability $P_{S_i,j}$ to visit an amenity multiplied by an important factor $\alpha$ as shown in Eq. 4. In this way, criticality for each system could have its importance factor like $\alpha _{health}$, or $\alpha _{education}$ to consider different systems. The important factor is a list of public services’ relevance provided by policymakers. Once the criticality for the node $C_{v_i}$ is computed, it is imputed to all edges $E_{ij}$ composing the trip from origin $S_i$ to *j*.

4 $$\begin{aligned} C_{v_i} = \alpha \cdot w_s \cdot P_{S_i,j} \end{aligned}$$

For instance, take two origins, A and C, in Fig. 2 to reach a healthcare facility and a school in node *H*. The computed criticality in node $C_{v_H}$ for the trip *AH* (in green) is $c_1$, and for the trip *CH* (in blue) is $c_2$. The criticality $c_1$ and $c_2$ affected edges $E_{AD}$, $E_{DE}$, $E_{EF}$, and $E_{CF}$, respectively. Finally, the multi-hazard criticality for segment $E_{F,H}$ will be the sum of both prior criticalities $c_1+c_2$. Once the scaled criticality of the road segments is estimated, we rank them to identify the most crucial road segments in the road network considering the exposure to both hazards.]

The last metric is the number of isolated people, which quantifies the nodes *V* without connection to the road network infrastructure *G* as the effect of road disruption, as shown in Eq. 5. Where the Boolean function *R* outputs one when destination *j* is not reachable from origin $S_i$ (*c.f.,* Eq. 6). Thus, *I* sums up the population that cannot reach any destination $w_s$.

5 $$\begin{aligned} I= & {} \sum _{S_i,h}^{S,H}{R(S_i,h) \times w_s } \end{aligned}$$

6 $$\begin{aligned} R(i,j)= & {} \left\{ \begin{array}{ll} 1, &{} {\text {if path from i to j } \not \exists } \\ 0, &{} else \end{array} \right. \end{aligned}$$

For example, in Fig. 2 if the edge $\overline{\textrm{AD}}$ connecting nodes *A* and *B* is disrupted, then the population assigned to node *A* becomes isolated.

Finally, the compound analysis is performed over the criticality and accessibility metrics. For the criticality of each edge *E* it is possible to add or take the maximum value for different hazards scenarios, while for accessibility, only the maximum function is applied. Therefore, based on the proposed methodology, we compare the difference between undisrupted (*G*(*V*, *E*)) and disrupted ($G_D(V,E)$) scenarios in terms of accessibility and criticality metrics to establish a risk profile for a single or compound hazard.

## Results

This section describes the health system accessibility and infrastructure criticality analysis results for the cities of Lima and Manila, two large and dense urban areas with complex health systems and road networks. In addition to the baseline case, our assessment considers disaster scenarios for floods (historical fluvial and pluvial floods), seismic fault lines, and compound hazards for each city’s 123 and 300 healthcare facilities, respectively. These factors are analyzed at the city block level, making both cities challenging case studies but crucial for actionable emergency preparedness planning. For both cases, we assumed a speed penalization factor of $\rho =3$ when a street intersects with a flood. We settle this value based on the fraction coefficient in the baseline scenario with congestion conditions ranging from 0.39 to 0.53, which means that speed decreases between 39% and 53%$^{47,48}$. Thus, in the flood hazards scenario, we assume that the road segment affected reduces its speed to a third of the speed under off-peak traffic conditions. While for fault lines, all streets within the buffer zone of 500*m* from the fault line are considered to be disrupted captured through additional delays due to closures(c.f., “Method” section). In addition, as a proxy of capacity, we use the amenities’ area $A_j= l_j \times w_j$ (where $l_j$ and $w_j$ are the length and width of the amenity area as shown in Fig. S1). For instance, a large educational amenity could have a kindergarten, elementary, middle, and high school. The same is true for healthcare; larger facilities offer more services than small ones. In the following section, we present the results of the performed experiments.

### Lima case study

**Figure 3:**
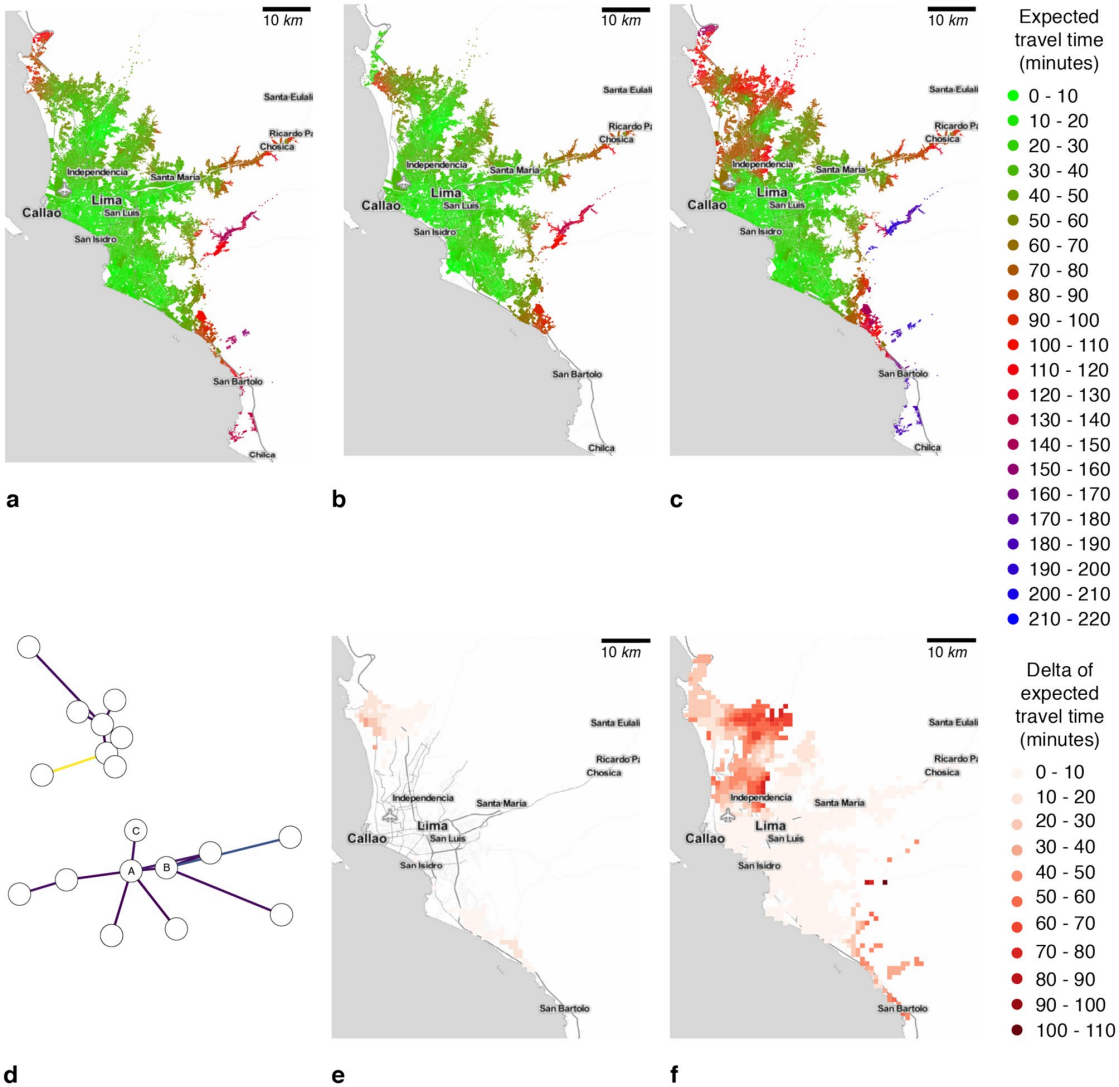
Accessibility map, at census unit scale, for healthcare facilities in Lima city. Where (**a**) is the undisrupted scenario, (**b**) is the earthquake scenario with a buffer of 500 m and a return period of 50 and 100 years, **(c)** is the flood scenario of flood planning hazard map at historical return period, (**d**) represents the number of nodes that shifted their preference from one healthcare facility to another as a graph, (**e**) is the heatmap of the accessibility difference between undisrupted and earthquake scenario with a return period of 50 and 100 years, and (**f**) is the heatmap of the accessibility difference between undisrupted and flood scenario of flood planning hazard map at historical return period, (**b**) shows an important accessibility time degradation in the north and in a small part in the south, (**c**) depicts a significant accessibility perturbation in the north of the city, and few changes in the south, (**d**) illustrates how healthcare provider preference change due to hazards, (**e**) shows an important accessibility time degradation in the north and in a small part in the south, (**f**) depicts a big accessibility perturbation in the north of the city and few changes in the south. Maps were created using Map Tiles by Stamen Design, CC BY 3.3 - Map data ©using Contextily 1.2.0 library for Python [maps.stamen.com].

Lima is Peru’s administrative and economic capital, with approximately ten million inhabitants. It is the third-largest city in Latin America, located on the Pacific coast and the Pacific Ring of Fire. In addition, Lima is built in the CHIRILU basin, composed of three rivers, the Chillón, the Rímac, and the Lurin, which originate in the Andes and make Lima vulnerable to flooding from upstream precipitation. The health network of Lima shows drastic differences in the number of healthcare facilities per district. The healthcare facility density per population is the highest in the city centre of Lima, and it decreases in more peripheral districts, except for the *Punta Hermosa* district in the south. Also, the city is greatly affected by congestion with a friction coefficient of 0.39, which means that average speed decreases by 39% in traffic jams^49^. Thus, even in the absence of any natural hazard affecting the transport network, the accessibility to health services varies drastically within the city, as shown in the analysis (*c.f.*, Fig. 3).

Regarding Lima’s risk profile, the considered hazards are floods and fault lines. We use official hazard maps with 20, 50, 100, and 500-year return periods to represent floods based on historic national fluvial floods data obtained from the Ministry of Environment^50^ (MINAM). Data on fault lines show historical seismicity, representing 50 and 100-year return periods, based on observations between 1960 and 2012 from Peru’s 33 seismogenic sources. The spatial distribution of such seismicity is associated with the subduction process (interface), the main fault systems (cortical) and the geometry of the Nazca tectonic plate (intraplate), as elaborated by the Geophysical Institute of Peru (IGP) and the Peruvian Geological, Mining and Metallurgic Institute^51^ (INGEMET). The average travel speeds on different road types are summarized on Table S2. Thus, we use these hazard maps to evaluate utility loss since alternative information about road exposure, vulnerability, and maintenance is not readily available or exists for modelling road network infrastructure’s fragility. In future, as information on fragility becomes available, it can be readily integrated into the model presented here.

Consequently, 18.8% of healthcare facilities and 28.9% of road network infrastructure are exposed to floods. For fault lines, the exposure drops to 5.5% of facilities and 6.2% of the infrastructure road network. Therefore, we evaluate a baseline scenario, a flood scenario, an earthquake scenario and a compound scenario where hazards overlap spatially and, in some cases, temporally. Such compound analysis allows us to perform a multi-risk assessment of different natural hazards.

First, the graph is built to model Lima’s road networks undirected graph *G*(*V*, *E*), where the vertices *V* are the 174, 887 street intersections and edges, *E* represent the 438, 468 streets. Accordingly, to reduce computation time, we randomly sample 5% of all nodes within a population density grid $G_{m5}$ (*c.f.*, Figure S2). We then use the road network graph $G_{m5}$ to compute the accessibility of undisrupted (baseline) and disrupted (earthquake, floods, and compound) scenarios, considering 3,391 origins and 123 healthcare facilities as destinations.

Consequently, the accessibility analysis in Fig. 3a depicts the average expected travel time to reach the healthcare system in the absence of hazards. From the Lima city center, the expected accessibility time is less than twenty minutes. However, it increases up to two hours in the urban periphery areas, such as Ancon, Santa Rosa, Lurigancho, Punta Negra, and San Bartolo districts – this is a direct result of healthcare facilities being mostly located in the city center.

For flood depths over 5 cm, the driving speed on affected links is reduced to one-third of the original speed. Exposed links to fault lines and deeper to 15 cm floods are fully disrupted^52^. Correspondingly, in the earthquake scenario depicted in Fig. 3b, we notice the increase from twenty minutes to one hour and a half of the expected travel time on Lima’s south side. The variation map in Fig. 3e highlights the highest accessibility decrease in Ancon, Santa Rosa, Puente Piedra, and Carabayllo districts in the north, and Lurin, Punta Hermosa, Punta Negra, and San Bartolo districts in the south. Concerning the historic flood scenario, 28.9% of healthcare facilities are directly exposed to floods. Aditionally, Fig. 3c illustrates the drastic increase in expected travel time from twenty minutes to three hours in Lurin, Punta Hermosa, Punta Negra, and San Bartolo southern districts; and one hour and a half in Ancon, Santa Rosa, Puente Piedra Carabayllo, San Martin, Comas, and Los Olivos (northern districts). Fig. 3f shows the accessibility changes due to floods, especially affecting the northern parts of Lima.

Since some routes are affected directly by the shock, the travel time to some healthcare facilities increases, reducing the facilities’ attractivity. Thus, populations change their healthcare facility preferences with respect to the baseline scenario as illustrated in the healthcare facility graph $G_H$ in Fig 3d. In this graph $G_H(V,E)$, the nodes *V* are the healthcare facilities, while edges *E* represent the number of healthcare preferences changed from one facility to another. For example, the edges capture the number of preferences that changed from healthcare facility A, in the undisrupted baseline scenario, to healthcare facility B, in the earthquake scenario. The darker the edge, the higher the number of preferences changed. For instance, 7.7% and 5.6% of the selected healthcare facilities (i.e., destinations) changed in the earthquake and flood scenarios, respectively.

**Figure 4:**
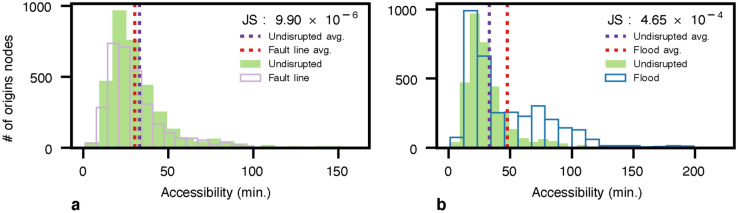
Accessibility time distribution to all reachable healthcare facilities in Lima city. Where (**a**) is the baseline and fault line scenario with a buffer of 500 m with a return period of 50 and 100 years; and (**b**) is the baseline and flood scenario of flood planning hazard map at historical return period. Flood in (b) illustrates a significant change in the accessibility time distribution concerning fault lines in (**a**). However, the number of accessible facilities reduces in (**a**) and the population’s healthcare facility priority list change. The Jensen-Shannon divergence are $9.9\times 10^{-6}$ and $4.65\times 10^{-4}$ for (a) and (b) concerning the undisrupted (baseline) scenario.

Additionally, Fig. 4 shows the accessibility distribution of earthquake and flood scenarios compared to the undisrupted (baseline) scenario. The accessibility here is the average expected travel time from each node to all healthcare facilities, providing a variety of health services based on an “attractiveness” ranking. To measure the impact of the hazards, we compute the Jensen-Shannon (*JS*) divergence for measuring the similarity between accessibility distributions in the presence and absence of hazards. We observe that the average expected travel time slightly decreases in the earthquake scenario, as depicted in Fig. 4a. This occurs due to infrastructure functionality loss in terms of lost and delayed trips and changes in facilities’ attractiveness. Losing access to some facilities and preferring the closer ones alter the average distance to reach a healthcare facility, from 9.8 Km. in the baseline scenario to 10.7 Km. and 9.6 Km. in fault line and flood scenarios. Note that for the Lima fault line scenario, 3.8% of original trips failed as longer trips are more likely to be disrupted, reducing the average accessibility time from 32.97 to 30.14 minutes. We also note a very small *JS* value of $9.9\times 10^{-6}$. Furthermore, regarding the flood hazard in Fig. 4b, we note that travel times significantly increase, from 32.97 to 47.68 minutes on average, as trips are possible but delayed, with a *JS* value of $4.65\times 10^{-4}$.

Baseline estimates suggest that 97.4% of the population can travel to a healthcare facility in under an hour. Then, 2.45% of inhabitants commute between one and two hours to attain a healthcare facility. Finally, the 0.12% population needs to move for more than two hours to arrive at a healthcare facility in an undisrupted scenario. Regarding the fault lines and flood hazard scenarios, we note a decrease in the number of individuals available to reach a healthcare facility in less than an hour to 93.45% and 78.18%, respectively. On the other side, we notice an increase in the population to 2.71% and 21.34%, for arriving at a healthcare facility between one and two hours, while for the extreme scenario, there is an increase of inhabitants to 0.38% due to flood. Finally, we observe that 3.8% of inhabitants could not reach the healthcare system due to road fracture in the fault line scenario, as summarized in Table 1. Finally, we remark that accessibility is compromised, and the impacts are concentrated on the city’s north, south, and east, while the historic city center is less affected.

**Table 1:** Population accessibility variation in undisrupted and disrupted (flood and fault lines) scenarios for Lima city.

hours	Undisrupted	Fault line	Floods
0 - 1	97.43%	93.45%	78.18%
1 - 2	2.45%	2.71%	21.34%
2 - 3	0.12%	0.04%	0.38%
3 - 4	0%	0%	0.10 %
Unreachable	0%	3.80%	0%

**Figure 5:**
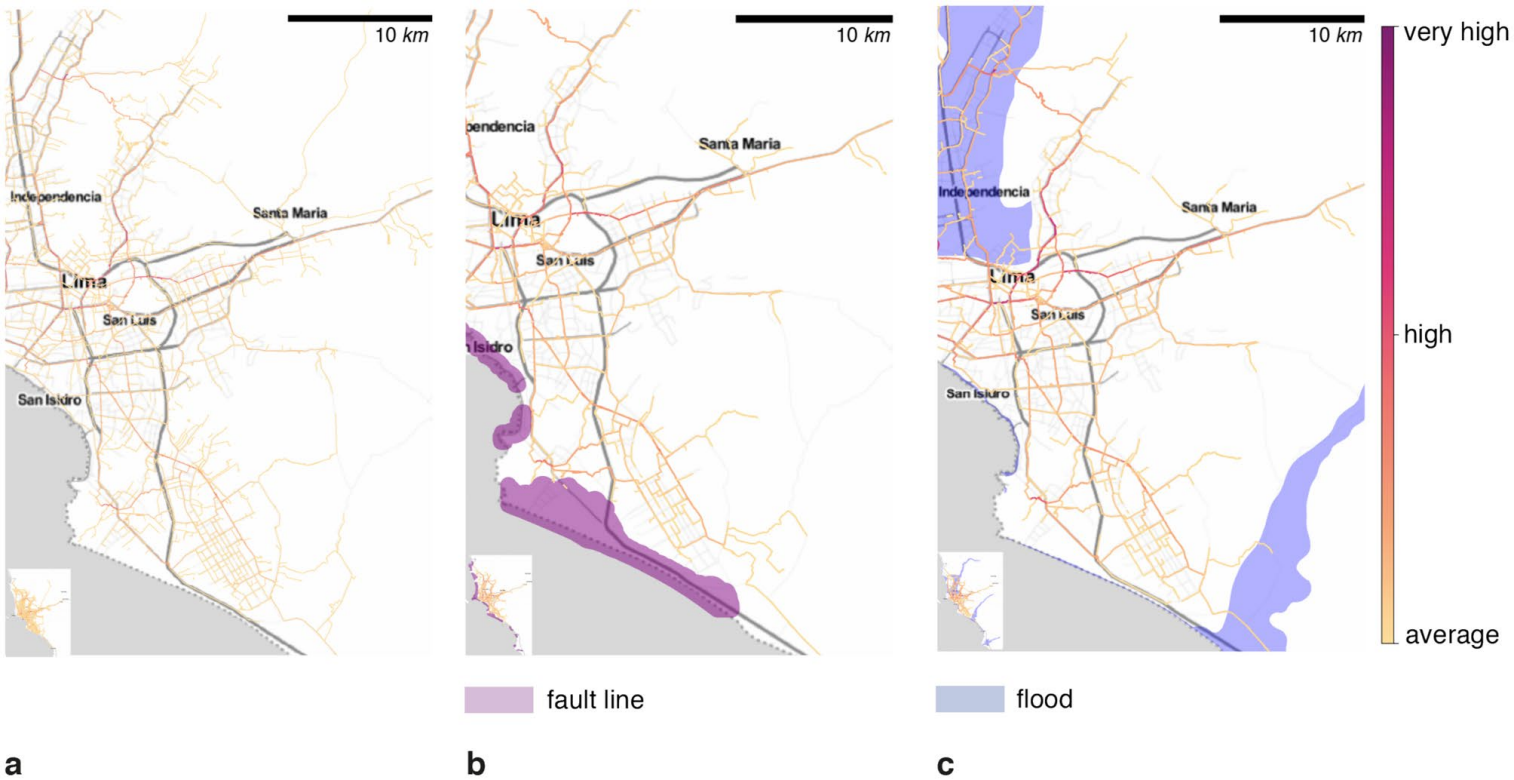
Criticality map for Lima road network infrastructure. Where (**a**) is the criticality in the undisrupted scenario, (**b**) is the criticality in the fault line scenario with a buffer of 500 m with a return period of 50 and 100 years, and (**c**) is the is the criticality flood scenario of flood planning hazard map at historical return period. (**b**) depicts the highest criticality in the main highway joining Lima north and south. (c) evinces a homogeneous degradation. Maps were created using Map Tiles by Stamen Design, CC BY 3.3 - Map data ©using Contextily 1.2.0 library for Python [maps.stamen.com].

**Figure 6:**
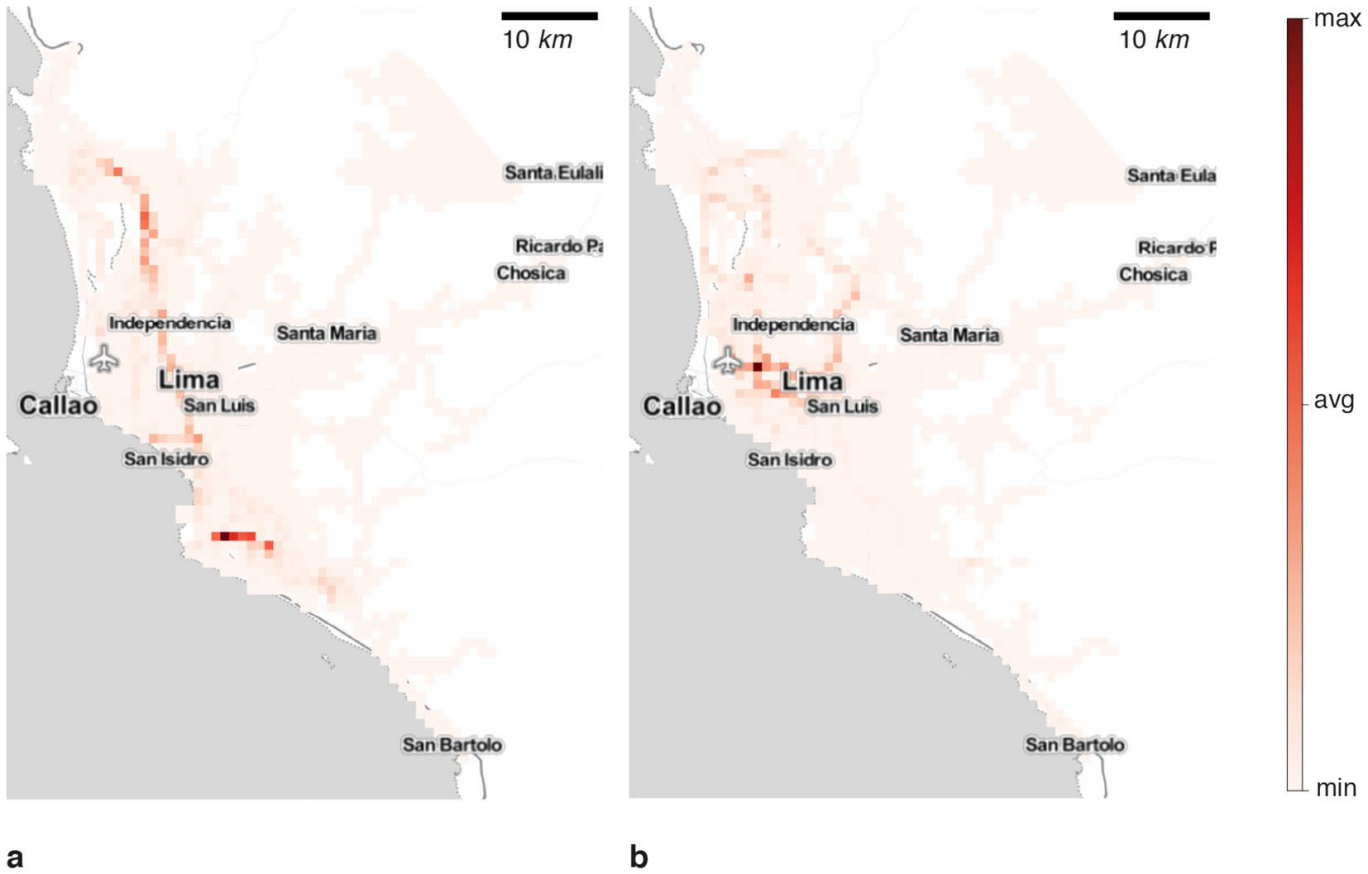
Road infrastructure criticality variation map for Lima. Where (**a**) is the criticality variation in the employed fault line hazard map with a disruption buffer zone of 500 m with a return period of 50 and 100 years, and (**b**) is the criticality variation in the employed flood hazard map of flood planning hazard map at historical return period. (**a**) depicts the highest criticality in the main highway joining Lima north and south. (b) evinces a homogeneous degradation. Maps were created using Map Tiles by Stamen Design, CC BY 3.3 - Map data ©using Contextily 1.2.0 library for Python [maps.stamen.com].

For assessing the important segments in the road network, we build a heatmap ranging from yellow (least critical) to red (most critical), as illustrated in Fig. 5. In these maps, the weights represent the number of times individuals passed through a road segment to reach a point of interest (i.e., healthcare facility).

**Figure 7:**
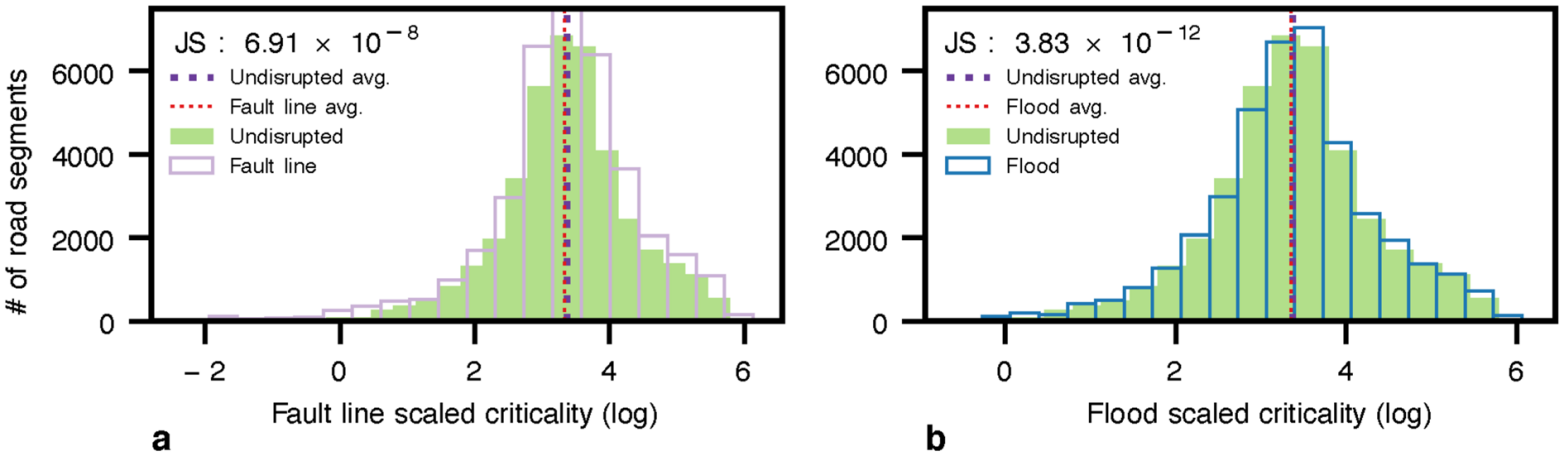
Criticality distribution for Lima city in log scale. Where (**a**) is the baseline and fault line scenario with a buffer of 500 m with a return period of 50 and 100 years, and (**b**) is the baseline and flood scenario of flood planning hazard map at historical return period. (a) show a slight change in the distribution. The Jensen-Shannon divergences are $6.91\times 10^{-8}$ and $3.83\times 10^{-12}$ for (a) and (b), respectively.

In our simulations, we limit the analysis to the healthcare system. Therefore, in the absence of disruption, the most critical axis to reach healthcare facilities is the *República de Panama Ave.*, which is the main highway of Lima city, connecting the city from north to south (Fig. 5a). Concerning the fault line scenario in Fig. 5b, we observe a small change in the main axis i.e., República de Panama Ave as detailed in Fig. 6a. To confirm this finding, we observe a modest switch between the scaled criticality distribution in the fault line scenario in Fig. 7a. Regarding the flood hazard, Fig, 5c depicts how the criticality segment change from the *República de Panama Ave.* in the north to the *Perú Ave.* in the north-east due to the river flood as depicted in Fig. 6b. Accordingly, the criticality distribution in Fig, 7b corroborates this fluctuation. In addition, the *JS* values for each scenario are $6.91\times 10^{-8}$ and $3.83\times 10^{-12}$, respectively. In addition to the maps, the model outputs the ID, and name of the segments or streets sorted by highest criticalities for the undisrupted (baseline), fault lines, and 5 cm flood depth scenarios in a fine grain level as shown in Table S3.

**Figure 8:**
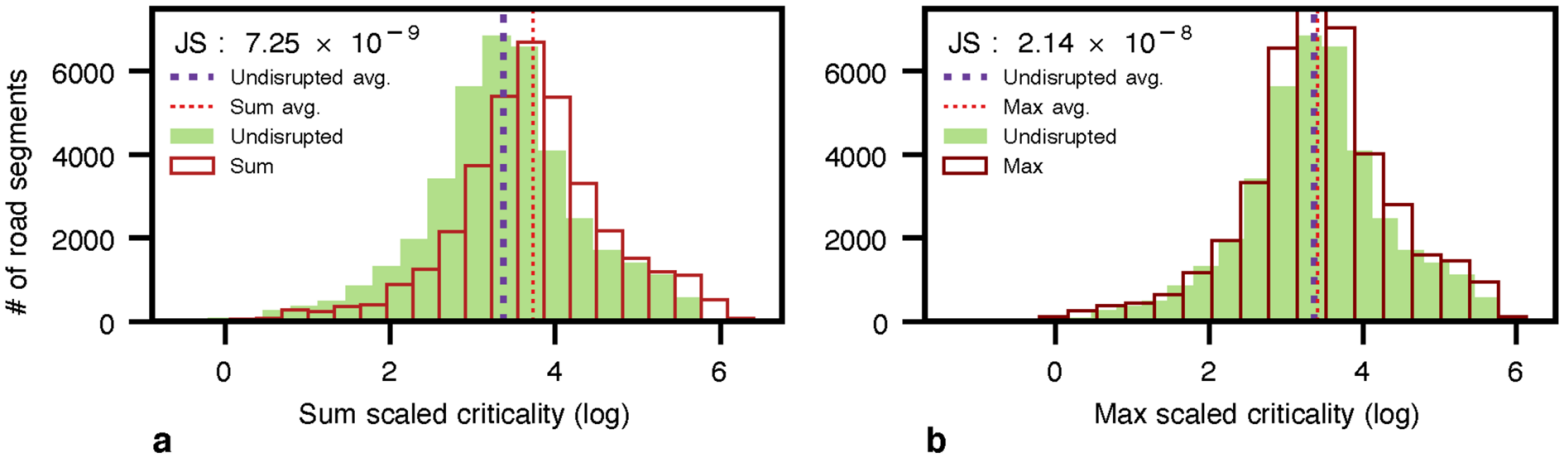
Criticality distribution for compound hazard fault line and flood for Lima city in log scale. Where **(a)** is the baseline and the compound hazard flood of flood planning hazard map at historical return period and fault line with a return period of 50 and 100 years taking the sum of both values, and **(b)** is the baseline and the compound hazard flood of flood planning hazard map at historical return period and fault line with a return period of 50 and 100 years taking the maximal value. The Jensen-Shannon divergences are $7.25\times 10^{-9}$ and $2.14\times 10^{-8}$ for (a) and (b) with respect to the undisrupted scenario.

In addition, we have considered a compound scenario where the floods and fault lines overlap spatially and, in some cases, temporally. Thus, we measure the criticality in this compound scenario in two ways. The former sums the flood and fault line criticalities per each segment in both scenarios, as depicted in Fig. 8a. The latter takes the maximal value between flood and fault line criticalities as illustrated in Fig. 8b. We note a substantial change in average criticality for the compound scenario when the sum is taken, as shown in Fig. 8a. At the same time, the increase of the average criticality is slightly more significant when taking the maximal value, as illustrated in Fig. 8b. Hence, the *JS* divergence are $7.25\times 10^{-9}$ and $2.14\times 10^{-8}$ for the sum and max scenarios compared to the undisrupted (baseline) scenario. The compound criticality map is available in Fig. S4 in the supplementary materials. In addition, Table S4 details the ID and name of the top ten road segments with the highest criticality in compound scenarios.

### Manila case study

Located in the east of the Philippines, Manila, the capital city, is the most densely populated city globally and is crossed by the Pasig, Marikina, and San Juan rivers. The city is divided into six legislative districts and sixteen geographic districts.

With respect to Manila’s risk profile, 3.78% and 6.96% of the healthcare facilities and infrastructure are exposed to fault lines with a return period of 500 years corresponding to the 1863 earthquake provided by the Philippine Institute of Volcanology and Seismology (*c.f.*, https://hazardhunter.georisk.gov.ph). In the flood scenario, at 5 cm 30.12% and 33.43%, and at 15 cm 14.33% and 16.16% of the healthcare facilities and road infrastructure are concerned, respectively. The return period, of the hazard maps provided by Fathom Global (*c.f.*, www.fathom.global), considered for 5 Cm. depth and 15 Cm. depth are 100 years. For Manila, we evaluate a baseline scenario, a flood scenario of 5 Cm. depth and 15 Cm. depth, a fault line scenario, and two compound scenarios where hazards overlap spatially and, in some cases, temporally. It is worth noting that compound analysis allows us to perform a multi-risk assessment of different natural hazards. For instance, the Philippines has a risk profile combining earthquake, wind, and flood hazards^37^. Therefore, the hazard maps are used as a proxy to evaluate utility loss since the information about exposure, vulnerability, and maintenance does not exist for modelling the fragility of the road network infrastructure.

**Figure 9:**
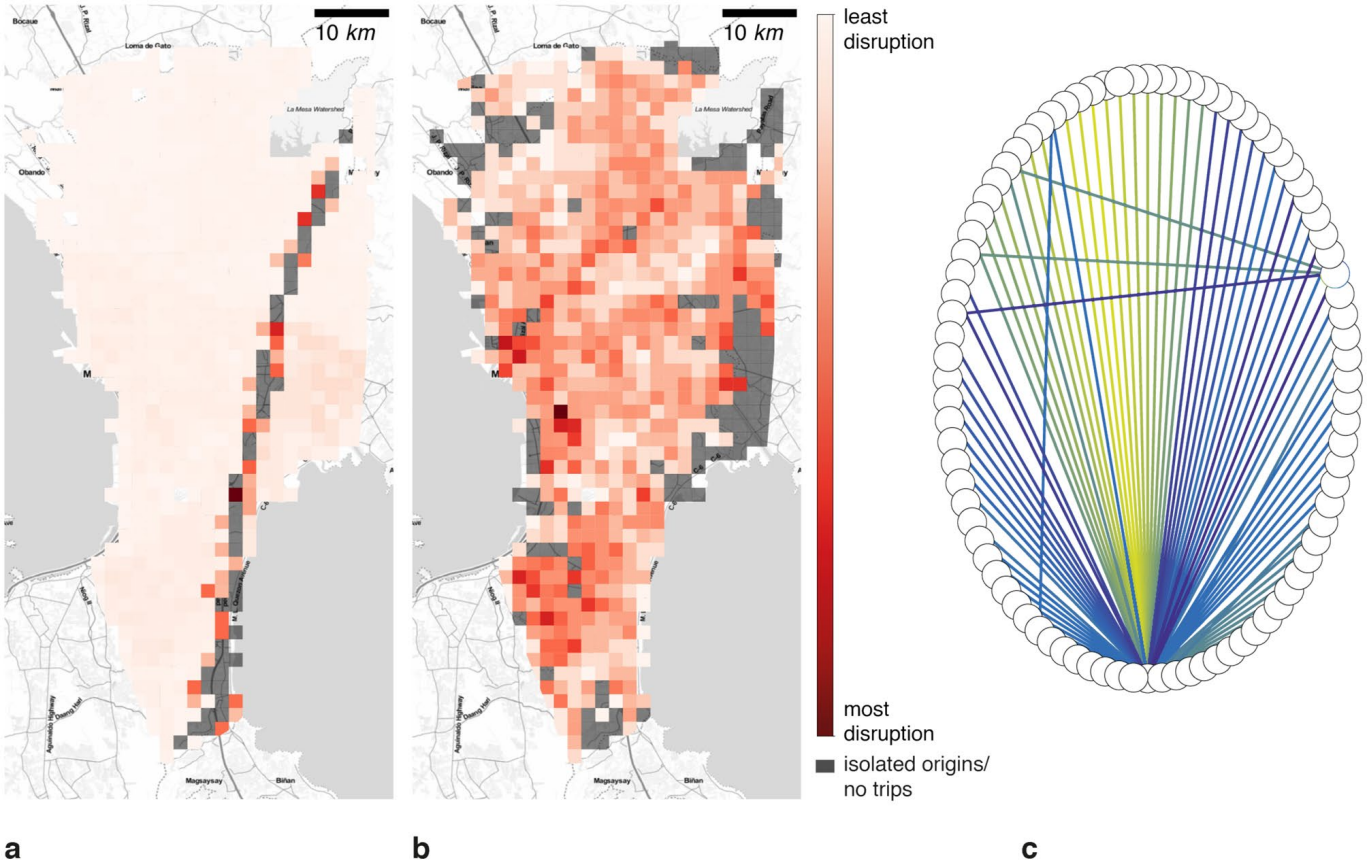
Isolated population map for Manila city. Where (**a**) is the fault line scenario with a return period of 500 years, (**b**) is the flood scenario with a return period of 100 years, and (**c**) is the healthcare facilities’ graph. (**a**) illustrates the isolated points over the West Valley fault. (**b**) Shows the isolated points in the boundaries of Metro Manila. (**c**) shows the decision change of healthcare facilities. Maps were created using Map Tiles by Stamen Design, CC BY 3.3 - Map data ©using Contextily 1.2.0 library for Python [maps.stamen.com].

Following the proposed methodology, Manila’s graph *G* containing 52 521 nodes and 89 183 edges is sub-sampled to consider around 5% of nodes as origins within the population grid of $1 Km \times 1 Km$. After the sub-sampling, we consider 2 202 origins for 300 healthcare facilities as destinations. Accordingly, to evaluate the accessibility of Manila’s healthcare facilities, we have considered three different hazard scenarios, namely fault line, flood at 5 cm, and flood at 15 cm. Figure S5 depicts the accessibility time change concerning the hazards. For instance, in the undisrupted scenario, in Fig. S5a, the accessibility time in the city center is up to 20 minutes. Then, it decreases to one hour around the city center until one hour and a half in the north and south of the Metro Manila. Some origin points in the city’s south have an accessibility time longer than two hours. Concerning the fault line scenario in Fig. S5b, we note a segment of the West Valley Fault increasing the accessibility time range from 40 minutes to one hour until two hours and a half in the east of Manila. In addition, this fault line isolates 7.28% of the population from the health system, which makes it impossible for them to reach a healthcare facility (see Table S5). Figure S6a shows the region with the accessibility variation due to the fault line. Regarding isolated people, Fig. 9a shows the map where isolated origins are located. We note the isolated points over the West Valley Fault.

As regards flooding, Figs. S5c and S5d illustrate the impact of floods at 5 cm and 15 cm. For instance, at 5 cm flood the population is not isolated, while at 15 cm 22.30% of the Manila population is isolated from the health system (see Table S5). Additionally, we note that accessibility time increases in the 5 cm flood scenario from 20 minutes to one hour and a half in a homogeneous way over the city as portrayed in Fig. S6b. On the contrary, in the 15 cm scenario, the remaining 77.55% of the population is able to reach a healthcare facility within 20 minutes when the 22.45% is isolated. However, the number of trips which can take place is reduced as some trips are fully disrupted. Nonetheless, there is an accessibility time increment in the west part of the city, as shown in Fig. S6c. Concerning isolated individuals, Fig. 9b illustrates the isolated origins, which are mostly on Manila’s borders.

Finally, the expected accessibility time distribution in Fig. S7 captures the general change of accessibility time where the *JS* divergences are 0.01, 0.002, and 0.018 for fault line, flood at 5 cm and flood at 15 cm with respect to undisrupted scenario. Thus, trips can take up to 75 minutes in the undisrupted scenario. However, accessibility time decreases due to the isolation of the east part of Manila in the fault line scenario and increases up to 100 minutes in flood scenarios. However, the average accessibility time for the flood at 15 cm decreases as illustrated in Fig. S7b. This behavior is expected since the disrupted trips are no longer present and the destination change as depicted in Fig. 9c, where the nodes are the healthcare facilities and edges represent the preference change from one healthcare facility to another. The darker the edge, the higher the number of preferences switch. Thus, the percent of changed destinations are 44.46%, 26.16%, and 35.1% for the fault line, floods at 5 cm and at 15 cm, respectively. In addition, the average travelled distance decreases from 15.3 Km. in the baseline scenario to 8.5 Km., 9.7 Km., and 12.68 Km. for the fault line, floods at 5 cm. and at 15 cm.

Concerning criticality estimation, Fig. S8 depicts the most used routes to reach the healthcare system through the transport infrastructure in four different scenarios, such as undisrupted, fault line, flood at 5 cm, and flood at 15 cm. In addition, Fig. S9 shows the most affected routes due to the natural hazards. For example, Figs. S8b and S9a evince the criticality variation affects Manila, Pasay, Parañque, and Las Piñas in the west of the city and Quezon, Pasig, Pateros, and Taguig in the east. Apropos floods, on the one hand, Figs. S8c and S9b show a more homogeneous change in criticality affecting most of the districts in Metro Manila. On the other hand, Figs. S8d and S9c, taking out the isolated population, reveal a more focus criticality variation in Manila, Caloocan, and Quezon in the north and Taguig, Muntinlupa, Parañque, and Las Piñas in the south. Looking at the criticality distribution in Fig. S10, we note an increment of the criticality and the trips lost. In addition, the *JS* divergence are $1.93 \times 10^{-7}$, $3.02 \times 10^{-8}$, and $2.42 \times 10^{-7}$ for fault line, flood at 5 cm, and flood at 15 cm with respect to the undisrupted scenario. The average criticality increases in the presence of natural hazards.

In addition, we characterized the places in the city where people are isolated from the healthcare system. Hence, Fig. 9 shows isolated areas around the districts of Marikina, Quezon City, Pasig, Makati, Taguig, and Muntinlupa over the West Valley Fault for the fault line scenario. On the other side, for the flood scenario, excluded points are located in Malabon and Valenzuela in the north; Manila, and Pasay, in the west; Parañaque and Muntinlupa in the south; and Pasig, Marikina, and Quezon in the east.

Up to now, we have analyzed fault lines and flood risk independently. Nevertheless, the before-mentioned hazards could coincide. Thus, our tool allows assessing accessibility and criticality in compound hazards. Accordingly, Figs. S11a and S11b illustrate a reasonable accessibility time of up to 20 minutes in the centre of Metro Manila, which is reduced, up to 80 min, as we move away from the city centre. The accessibility distributions confirm these variations as illustrated in Fig. S12. Concerning the compound criticality, Fig S13 reveals the affected routes in the Manila, San Juan, Quezon, and Mandaluyong districts. Thus, the *JS* divergence are $6.98\times 10^{-8}$ and $1.45\times 10^{-7}$ for sum and max scenarios compared to the undisrupted scenario. Additionally, Table S6 details the ID and name of the top ten road segments with the highest criticality in compound scenarios.

## Discussion

In the present work, we have explored the need to build population accessibility and infrastructure criticality metrics based on services they provide in normal circumstances and facing single or multiple hazards. The accessibility metric quantifies how easy it is for the population to reach a given amenity, which provides a public service. At the same time, criticality determines the crucial road segments to reach important amenities. Thus, criticality can be extended to other infrastructures and services without loss of generality. The observed limitations on the construction of such indicators are data availability, accessibility, and feasibility of computations at an actionable level of detail. In the former case, the existing data is not publicly available for this kind of analysis, which is more penalizing for developing countries. In the latter case, the country’s national statistics office does not always create the needed data due to economic and geographic access limitations. Both situations cause limited studies in terms of accessibility and criticality concerning public services resilience. To overcome these problems, we proposed a methodology to quantify accessibility and criticality to different public services such as healthcare, education, *Etc.* using available open data and providing actionable outputs for stakeholders. This analysis is one of its kind in data-driving cross-sectoral investment prioritization.

Based on the literature review, we compare our approach to two different and widely used methods: the gravity model and the Two-step Floating Catchment Area Method (2SFCA). The former captures the interaction between health supply and demand in different locations using a decay function as distance^53^. The drawback of the gravity model is the number of needed inputs. For example, location of healthcare facilities, healthcare facilities’ healthcare offer or capacity, location of healthcare demand, road network, and travel time analysis between supply and demand^54^, which could be hard to find, especially in developing countries. The latter model (2SFCA) takes the population location, the number of physicians at each healthcare facility and travel distance matrix from population location to healthcare facility^55^. As mentioned before, these datasets could be complicated to gather due to information restrictions or existence. In the work of Hierink *et al.*^56^, they also measure accessibility, in minutes, to healthcare facilities using road network extract from OpenStreetMap. However, their analysis is coarser compared to ours and only considers children under five years old. At the same time, our approach considers the whole population and reports the criticality of the transport infrastructure. Additionally, the authors measure accessibility after a cyclone, whereas our study considers different hazards such as fault lines, floods and compound risk. Another study, the work of Ceferino *et al.*^40^ evaluates patient transference strategies after an earthquake in Lima, Peru. The authors represent the healthcare system as a set of 41 hospitals, while in our approach, we handle 123 healthcare facilities for both seismic and flood hazards. Regarding the individuals’ process of selecting their healthcare provider, we overcome the generalized hypothesis of choosing the closest facility^57^ by introducing an attractivity function that takes into account the healthcare capacity and the travel time. Consequently, our approach allows to identify: (1) population, (2) amenities (i.e., hospitals), (3) average accessibility time, and (4) critical infrastructure segments at risk. Moreover, it produces geospatial maps of accessibility and criticality.

In our proposal, the primary inputs are the road network and amenities’ locations from OpenStreetMap to assess accessibility and criticality. In addition, we can add population and hazards information for accessibility and criticality analysis in disrupted scenarios. Therefore, our metrics to quantify accessibility and criticality are easily interpretable. Our accessibility metric measures the time to access the health system considering individuals’ preferences in terms of treatment facility, type of services needed, and healthcare facility capacity and quality of care. The time here stands for broader access cost which entails travel costs as well as time lost by missing work or school which can be considered explicitly in our proposed methodology given the availability of data. Thus, our metric goes beyond existing approaches of measuring travel time within a catchment area of a single facility^20,23–25,27–30^ or to the closest facility^20,26^ without considering the type of facility or the services it provides. The latter has been proven to be a very strong assumption in the health-seeking behavior of the population. Our infrastructure criticality metric captures the relative priority of the road segments at the street level based on how many users are traveling through and for what purpose. The granularity of analysis is novel and crucial for investment prioritization compared to the existing body of work. Methodologically, we proposed and implemented the measure that is based on edge betweenness centrality weighted by the relative importance of the public services (health, education, etc.) and estimated travel density on the road segments based on users’ preferences based on accessibility, service types, capacity, and quality. Other criticality metrics in the literature rely on betweenness edge centrality weighted by simulated traffic flow^58^, Macroscopic Fundamental Diagram (MDF)^31^, economic loss based on products transportation in the road segments^32^, accessibility loss due to disruptions on the road^33^, and road section with high the traffic flow contribution^34^ to capture road criticality. Furthermore, other information such as capacity, number of services, human resources, *Etc.* could be added to the attractivity function of the Huff model for customizing the study. Finally, our method is designed to be parallel for optimizing computation time, especially when analyzing megacities.

It is important to note that even though we rely on Open Data, which could be incomplete, we are able to perform meaningful analysis of accessibility and criticality for different public services such as healthcare facilities under different scenarios like undisrupted, disrupted and compound disruption. We considered compound risk in this work is not primarily focused on simultaneous multiple hazards as we aim to capture the spatial exposures. We are making this case especially for hazard-agnostic investments in road networks such as routine maintenance. Here, we suggest prioritizing the road segments that are relatively more important in both hazards for consideration. For hazard-specific investments, such as flood protection interventions, the decision maker should refer to the related hazard-specific priority list. Also, for Manila, we added the low probability of having to prepare and respond to both hazards at the same time.

Our tool allows policymakers, Non-Governmental organizations, and individuals to perform a cross-sectoral investment to ensure the resiliency of public services. The end goal of our tool is to ensure resilient access to public services for the population, service providers, and the supply chain to enable continuous running of the services. Thus, these studies could help to understand the population’s needs regarding access to public services. Our methodology is a computationally parallel feasible method to identify the critical elements in the infrastructure network for resilient investments in urban areas. The methodology is based on the topology of the infrastructure network, public services, and population distribution. Furthermore, the proposed methodology enables policymakers to analyze different scenarios caused by natural hazards to assess the impact of such disruption over the road network in terms of time to reach public services and the usage of the road segments. Therefore, the proposed mechanism could be used to perform analysis in different cities worldwide to analyze the state of the accessibility and criticality of public services.

## Conclusion

In the current effort, we proposed a new methodology for computing urban accessibility to essential public services such as healthcare and infrastructure component criticality analysis regarding services they provide, such as transport, relying on open data, and less intensive computational cost. Additionally, this methodology enables the identification of isolated locations and populations regarding the studied public service. The analysis is granular enough to be of use to urban planning directly. The likelihood of choosing one service provider over another is captured in the model, expected access times for various services are estimated for population, and all the computation considers population and services. It is a scalable and modular method and tool that simulates different hazard scenarios like floods, fault lines, or exposure to compound risks enabling cross-sectoral decision-making in the health and infrastructure sectors concerning emergency planning and response. Moreover, our proposed work could be easily extended to analyze other public services such as education, finance, judiciary, *Etc.* and infrastructures like power, water and sanitation.

The proposed methodology was applied to compute the expected accessibility time to all services depending on the country’s risk profile to natural hazards In Lima and Manila. For instance, in Lima, 4% of the population increase their accessibility time by more than an hour in the fault lines scenario, while for floods, the number increases to 19.25%. The methodology allows us to capture when population preference shifts to closer facilities for health services in a fault lines scenario. In Manila city, hazards like fault lines, floods at 5 cm and floods at 15 cm push 21.36%, 14.35%, and 23.1% of the population above 60 minutes threshold, respectively. These measures are for the population who still have access, but some individuals can no longer reach any health services during shocks. For example, for fault lines, 3.80% and 7.28% of the population become isolated in Lima and Manila, respectively. As Manila is more susceptible to floods, 22.30% of the population loses access to higher health services in case of heavier floods. The method identifies the hazard affecting more people, the location, and the percentage of isolated individuals while adapting to people’s choices. On infrastructure criticality, we provide maps and names of critical road segments to ensure accessibility to public services. On aggregated level, we show the average criticality changes for functioning links.

The proposed criticality analysis method considers the public service of choice and the desired combination of services. That is, the most critical segments to ensure access of the most people to health services is computed. Therefore, policymakers could use the names, locations and metrics to prioritize cross-sectoral investments to improve the resiliency of public services and the supporting lifelines.

In addition, our methodology captures the individuals’ healthcare service provider change due to infrastructure exposure to hazards. Thus, the Huff model shows how healthcare facilities’ attractivity changes. Accordingly, 7.67% and 5.6% of the selected healthcare facilities change in Lima’s fault line and flood scenarios. Regarding Manila, the percent of changed destinations are 44.46%, 26.16%, and 35.1% for the fault line floods at 5 cm and at 15 cm, respectively.

Finally, the new research avenues will be to consider the effect of traffic congestion on accessibility, to couple more attractivity parameters for the Huff model to capture people’s choices and to add more information layers like socio-economic factors to the population information for more in-depth analysis of vulnerable populations.

## Supplementary Information

Supplementary Information.

## Data Availability

In the present work, the datasets for building the road network were extracted from *OpenStreetMaps*^59^, which is a publicly available source. We used the Python package OSMnx^42^ to download the geospatial data from *OpenStreetMaps*. For the population density, we rely on the *WorldPop* project^60^ that provides global human population counts. The flood^50^, and fault lines^51^ datasets for Peru were downloaded from the GPS Peru site. Finally, the dataset for Manila hazards was gathered from the Global fluvial and surface flood hazard data (May 2017 version), which was used with the permission of Fathom Global. Please note that maps source data are provided as a Source Datafile, and the base map layer is available under a https://www.openstreetmap.org/copyright Open Database Licence ($\copyright$OpenStreetMap Contributors). The code and data sources to replicate Lima results is available at: https://dataverse.harvard.edu/privateurl.xhtml?token=2697a3c6-8fef-489c-809d-bc9c364cf66e.

## References

[1] Deziel NC (2022). Assessing community-level exposure to social vulnerability and isolation: Spatial patterning and urban-rural differences. J. Expo. Sci. Environ. Epidemiol..

[2] Thacker S (2019). Infrastructure for sustainable development.. Nat. Sustain..

[3] Alatrista-Salas H, Gauthier V, Nunez-del Prado M, Becker M (2021). Impact of natural disasters on consumer behavior: Case of the 2017 el niño phenomenon in Peru. PloS One.

[4] Fan C, Jiang X, Lee R, Mostafavi A (2022). Equality of access and resilience in urban population-facility networks. npj Urb. Sustain..

[5] Kaplan KH (2020). Accessibility to emergency food systems in south-central indiana evaluated by spatiotemporal indices of pressure at county and pantry level. Nat. Food.

[6] Bauer J, Klingelhöfer D, Maier W, Schwettmann L, Groneberg DA (2020). Spatial accessibility of general inpatient care in Germany: An analysis of surgery, internal medicine and neurology. Sci. Rep..

[7] Christodoulou A, Dijkstra L, Christidis P, Bolsi P, Poelman H (2020). A fine resolution dataset of accessibility under different traffic conditions in European cities. Sci. Data.

[8] Petri G, Expert P, Jensen HJ, Polak JW (2013). Entangled communities and spatial synchronization lead to criticality in urban traffic. Sci. Rep..

[9] Hamedmoghadam H, Jalili M, Vu HL, Stone L (2021). Percolation of heterogeneous flows uncovers the bottlenecks of infrastructure networks. Nat. Commun..

[10] Wang W, Yang S, Stanley HE, Gao J (2019). Local floods induce large-scale abrupt failures of road networks. Nat. Commun..

[11] Loreti S, Ser-Giacomi E, Zischg A, Keiler M, Barthelemy M (2022). Local impacts on road networks and access to critical locations during extreme floods. Sci. Rep..

[12] Danziger MM, Barabási A-L (2022). Recovery coupling in multilayer networks. Nat. Commun..

[13] Korkali M, Veneman JG, Tivnan BF, Bagrow JP, Hines PD (2017). Reducing cascading failure risk by increasing infrastructure network interdependence. Sci. Rep..

[14] Gastner MT, Newman ME (2006). Optimal design of spatial distribution networks. Phys. Rev. E.

[15] Neutens T (2016). Accessibility, in transportation planning. Int. Encycl. Geogr. People Earth Environ. Technol. People Earth Environ. Technol..

[16] Neutens T, Delafontaine M, Scott DM, De Maeyer P (2012). A gis-based method to identify spatiotemporal gaps in public service delivery. Appl. Geogr..

[17] Qin J, Liu Y, Yi D, Sun S, Zhang J (2020). Spatial accessibility analysis of parks with multiple entrances based on real-time travel: The case study in beijing. Sustainability.

[18] Barboza MH, Carneiro MS, Falavigna C, Luz G, Orrico R (2021). Balancing time: Using a new accessibility measure in Rio de Janeiro. J. Transp. Geogr..

[19] Nunez-del Prado, M. & Barrera, J. Analysis of the health network of metropolitanlima against large-scale earthquakes. In *7th Annual International Conference SIMBig 2020* (2020).

[20] Kang J-Y (2020). Rapidly measuring spatial accessibility of covid-19 healthcare resources: A case study of Illinois, USA. Int. J. Health Geogr..

[21] Levinson D, Wu H (2020). Towards a general theory of access. J. Transp. Land Use.

[22] Jiao J, Moudon AV, Ulmer J, Hurvitz PM, Drewnowski A (2012). How to identify food deserts: Measuring physical and economic access to supermarkets in king county, Washington. Am. J. Public Health.

[23] Wang J, Du F, Huang J, Liu Y (2020). Access to hospitals: Potential vs. observed. Cities.

[24] Luo W, Wang F (2003). Measures of spatial accessibility to health care in a Gis environment: Synthesis and a case study in the Chicago region. Environ. Plan. B Plan. Des..

[25] Kiani B, Mohammadi A, Bergquist R, Bagheri N (2021). Different configurations of the two-step floating catchment area method for measuring the spatial accessibility to hospitals for people living with disability: A cross-sectional study. Arch. Public Health.

[26] Zhao P, Li S, Liu D (2020). Unequable spatial accessibility to hospitals in developing megacities: New evidence from Beijing. Health Place.

[27] Qian T, Chen J, Li A, Wang J, Shen D (2020). Evaluating spatial accessibility to general hospitals with navigation and social media location data: A case study in Nanjing. Int. J. Environ. Res. Public Health.

[28] Boisjoly G (2020). Measuring accessibility to hospitals by public transport: An assessment of eight Canadian metropolitan regions. J. Transp. Health.

[29] Zhou X, Yu Z, Yuan L, Wang L, Wu C (2020). Measuring accessibility of healthcare facilities for populations with multiple transportation modes considering residential transportation mode choice. ISPRS Int. J. Geo-Inf..

[30] Tao Z, Cheng Y, Du S, Feng L, Wang S (2020). Accessibility to delivery care in Hubei province, China. Soc. Sci. Med..

[31] Kim S, Yeo H (2017). Evaluating link criticality of road network based on the concept of macroscopic fundamental diagram. Transportmetrica A: Transp. Sci..

[32] Colon C, Hallegatte S, Rozenberg J (2021). Criticality analysis of a country’s transport network via an agent-based supply chain model. Nat. Sustain..

[33] García-Palomares JC, Gutiérrez J, Martín JC, Moya-Gómez B (2018). An analysis of the Spanish high capacity road network criticality. Transportation.

[34] Su F, Zou X, Qin Y, She S, Su H (2020). Critical section identification in road traffic network based on spatial and temporal features of traffic flow. Green, Smart and Connected Transportation Systems.

[35] Wang F (2003). Job proximity and accessibility for workers of various wage groups. Urb. Geogr..

[36] He Y, Thies S, Avner P, Rentschler J (2021). Flood impacts on urban transit and accessibility-a case study of Kinshasa. Transp. Res. Part D: Transp. Environ..

[37] Nassirpour, A., Galasso, C. & D’Ayala, D. Multi-hazard physical vulnerability prioritization of school infrastructure in the philippines. In *11th National Conference on Earthquake Engineering 2018, NCEE 2018: Integrating Science, Engineering, and Policy*, vol. 10, 6456–6467 (Earthquake Engineering Research Institute, 2018).

[38] Depietri Y, Dahal K, McPhearson T (2018). Multi-hazard risks in New York city. Nat. Hazards Earth Syst. Sci..

[39] Safaripour M, Monavari M, Zare M, Abedi Z, Gharagozlou A (2012). Flood risk assessment using gis (case study: Golestan province, Iran). Pol. J. Environ. Stud..

[40] Ceferino L, Mitrani-Reiser J, Kiremidjian A, Deierlein G, Bambarén C (2020). Effective plans for hospital system response to earthquake emergencies. Nat. Commun..

[41] Ramirez-Rubio O (2019). Urban health: An example of a "health in all policies" approach in the context of sdgs implementation. Glob. Health.

[42] Boeing G (2017). Osmnx: New methods for acquiring, constructing, analyzing, and visualizing complex street networks. Comput. Environ. Urb. Syst..

[43] Map, O. S. (2014) Open street map. *Retriev. March* **18**, 2014

[44] World Bank. GOSTNets build, process, and analyze networks. https://github.com/worldbank/GOSTnets. Online; accessed 10 May (2021).

[45] Rocchi A (2022). A machine learning framework for multi-hazard risk assessment at the regional scale in earthquake and flood-prone areas. Appl. Sci..

[46] Jones JN, Boulton SJ, Stokes M, Bennett GL, Whitworth MR (2021). 30-year record of Himalaya mass-wasting reveals landscape perturbations by extreme events. Nat. Commun..

[47] Nunez-del Prado, M. & Barrera, J. (2020) Analysis of the health network of metropolitan lima against large-scale earthquakes. In *Annual International Conference on Information Management and Big Data*, 445–459 Springer.

[48] Huff DL (1963). A probabilistic analysis of shopping center trade areas. Land Econ..

[49] Rozenberg, J., Briceno-Garmendia, C., Lu, X., Bonzanigo, L. & Moroz, H. Improving the resilience of peru’s road network to climate events. *Proc. Nat. Academy Sci.* (2017).

[50] Juan Suyo. Mapa de Áreas inundables del ministerio de medio ambiente-susceptibilidad shapefile and kmz-geo gps perÚ. https://www.geogpsperu.com/2020/01/mapa-de-areas-inundables.html (2020). Accessed: 2022-04-11.

[51] Juan Suyo. Fallas geológicas del perú del instituto geológico, minero y metalúrgico shapefile - geo gps perÚ. https://www.geogpsperu.com/2020/07/fallas-geologicas-del-peru-descargar.html (2020). Accessed: 2022-04-11.

[52] Rentschler J, Salhab M, Jafino BA (2022). Flood exposure and poverty in 188 countries. Nat. Commun..

[53] Guagliardo MF (2004). Spatial accessibility of primary care: concepts, methods and challenges. Int. J. Health Geogr..

[54] Joseph, A. E. & Phillips, D. R. Accessibility and utilization: Geographical perspectives on health care delivery (1984).

[55] Radke J, Mu L (2000). Spatial decompositions, modeling and mapping service regions to predict access to social programs. Geogr. Inf. Sci..

[56] Hierink F, Rodrigues N, Muñiz M, Panciera R, Ray N (2020). Modelling geographical accessibility to support disaster response and rehabilitation of a healthcare system: An impact analysis of cyclones idai and kenneth in mozambique. BMJ open.

[57] Alabbad Y, Mount J, Campbell AM, Demir I (2021). Assessment of transportation system disruption and accessibility to critical amenities during flooding: Iowa case study. Sci. Total Environ..

[58] Ahmed MA, Kays H, Sadri AM (2023). Centrality-based lane interventions in road networks for improved level of service: the case of downtown boise, idaho. Appl. Netw. Sci..

[59] Map, O. S. Open street map. *https://www.openstreetmap.org* **15**, 30–0415 (2017).

[60] Tatem AJ (2017). Worldpop, open data for spatial demography. Sci. Data.

