# Supplementary material for: Measuring accessibility to public services and infrastructure criticality for disasters risk management
Source: Sci Rep. 2023 Jan 28;13:1569. doi: 10.1038/s41598-023-28460-z (PMC9884248; doi:10.1038/s41598-023-28460-z)
Supplement: Supplementary file 1 — Supplementary Information. [file 41598_2023_28460_MOESM1_ESM.pdf]

Supplementary Material

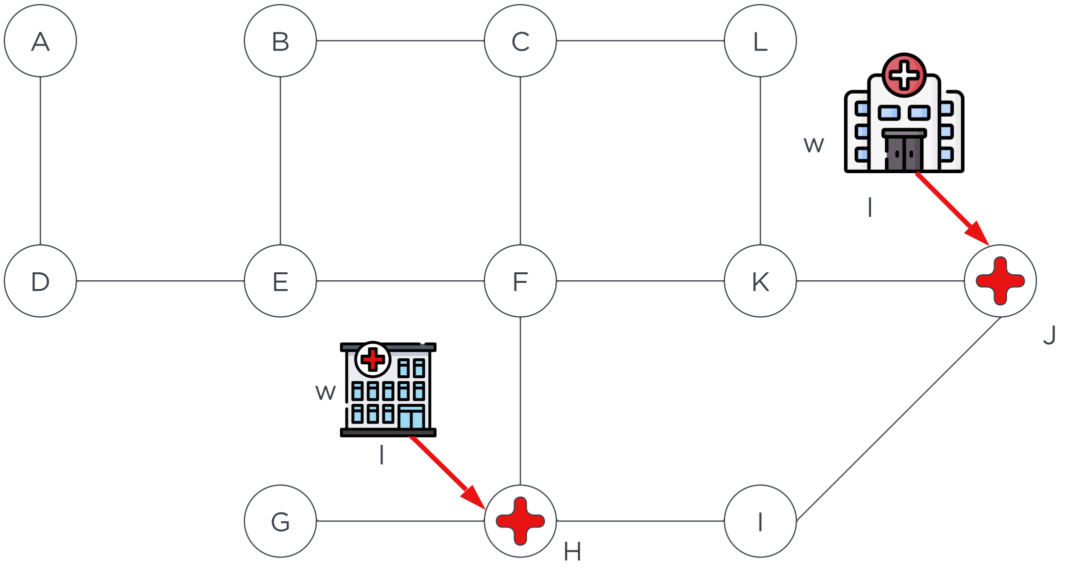

Figure S1. Snap example of healthcare facilities to a synthetic grap.

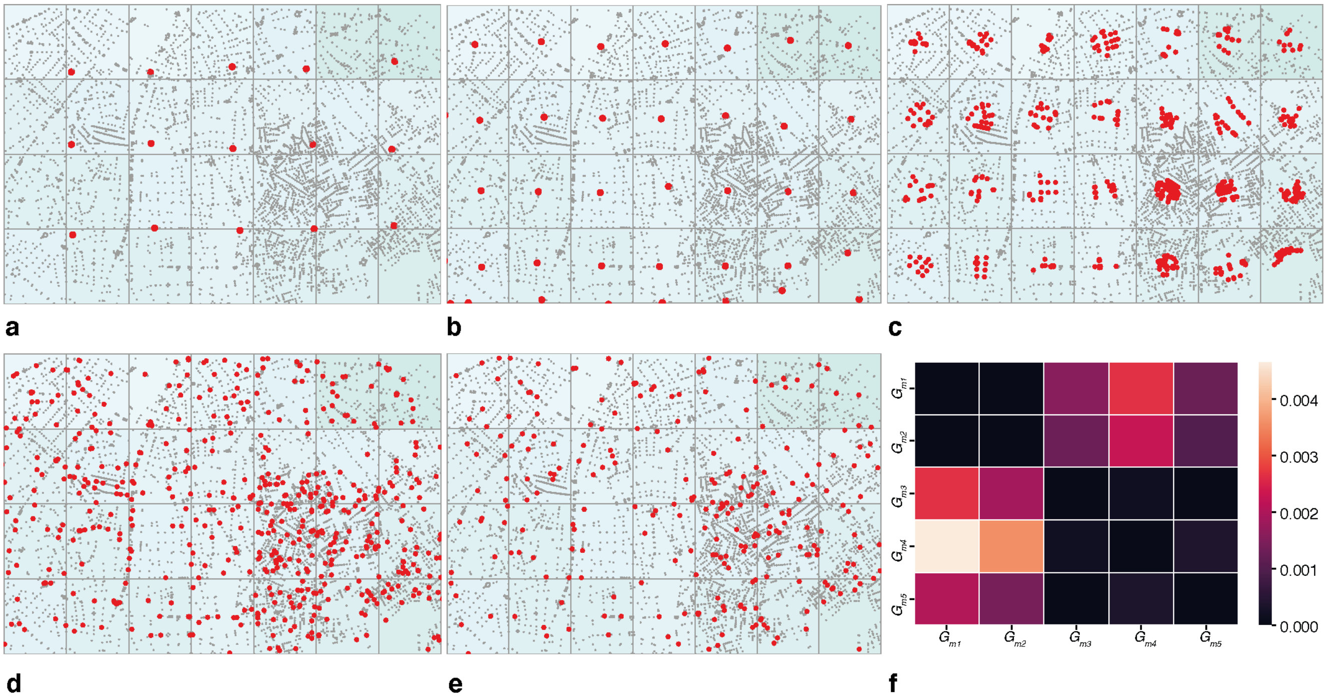

Figure S2. Different strategies to select origin points. **(a)** fixed grid of $1Km \times 1Km$ (G_{m1}); **(b)** centroids of a population density grid (G_{m2}); **(c)** 10% of closest nodes to the centroid node within each polygon defined by the population density grid (G_{m3}). **(d)** 10% of random points within a population density grid (G_{m4}); **(e)** 5% of random nodes within a population density grid (G_{m5}); and **(f)** Jensen–Shannon divergence of degree different distributions issued from the strategy to pick origin points. G_{m5}) shows a suitable trade-off between representative origins and divergence value.

Author	Model	Transportation mean	Study unit	Assumption	Origin	Destination	Road network	Speeds
Wang *et al.*[23]	"E2SFCA "	Taxi	1 × 1-km grid unit	Shortest travel time	Pick up points	General and specialized hospitals	OSM	Urban Road Engineering Design Specifications and the reality of Beijing
Tao *et al.*[30]	GV2SFCA	Private vehicle	Town level	shortest travel time	Centroids of towns or streets	Public hospitals providing obstetric service	Baidu	Fastest travel time
Zhao, Li, and Liu[26]	"E2SFCA (tertiary and secondary) and travel time to the nearest facility for primary and neighbourhood "	Public transport and private vehicle	Neighborhood	Travel time to the nearest facility	Neighborhood centroid	Tertiary, secondary, primary, and neighborhood hospital levels	Baidu	dynamic traffic information from Baidu
Qian *et al.*[27]	Ga2SFCA	Public transport and private vehicle	1 × 1-km grid unit	Shortest travel time	Grid centroid	General hospitals	Baidu	Dynamic traffic information from Baidu
Boisjoly *et al.*[28]	"2SFCA "	Public transport	Census tract	Time of the fastest route	Census tract	Census tract containing at least 1 hospital	Arcgis	Canadian General Transit Feed Specification
Zhou *et al.*[29]	MTM-RTMC G2SFCA	driving, walking, public transportation, and bicycling		Fastest way	Street block	Pediatric clinic	Amap Maps	Real time traffic condition from Amap Maps
Kang *et al.*[20]	"P-E2SFCA "	Driving	Hexagons	Travel time	Residential location	Hospitals	OSM	Fastest travel time
Alabbad *et al.*[20]	Dijkstra	Driving	Nodes	Accessibility to the closest critical amenity, in terms of shortest-path length	Nodes	Hospitals, fire departments, and police stations	OSM	Accessibility to the closest critical amenity
Kiani *et al.*[25]	E2SFCA	Driving	Neighborhood	Travel-time to the closest distance to hospitals	Neighborhood centroid	Hospitals	ArcMap	Closest distance to hospitals

Table S1. Accessibility works summary.

Road type	Speed in baseline scenario (Km/h)	Speed in hazard scenario (Km/h)
Residential	15	5
Primary	35	12
Primary link	30	10
Motorway	45	15
Motorway link	40	16
Trunk	35	12
Trunk link	30	10
Secondary	25	8
Secondary link	20	7
Tertiary	25	8
Tertiary link	20	7
Unclassified	15	5

Table S2. Average travel speeds on different road types for baseline and hazard scenarios.

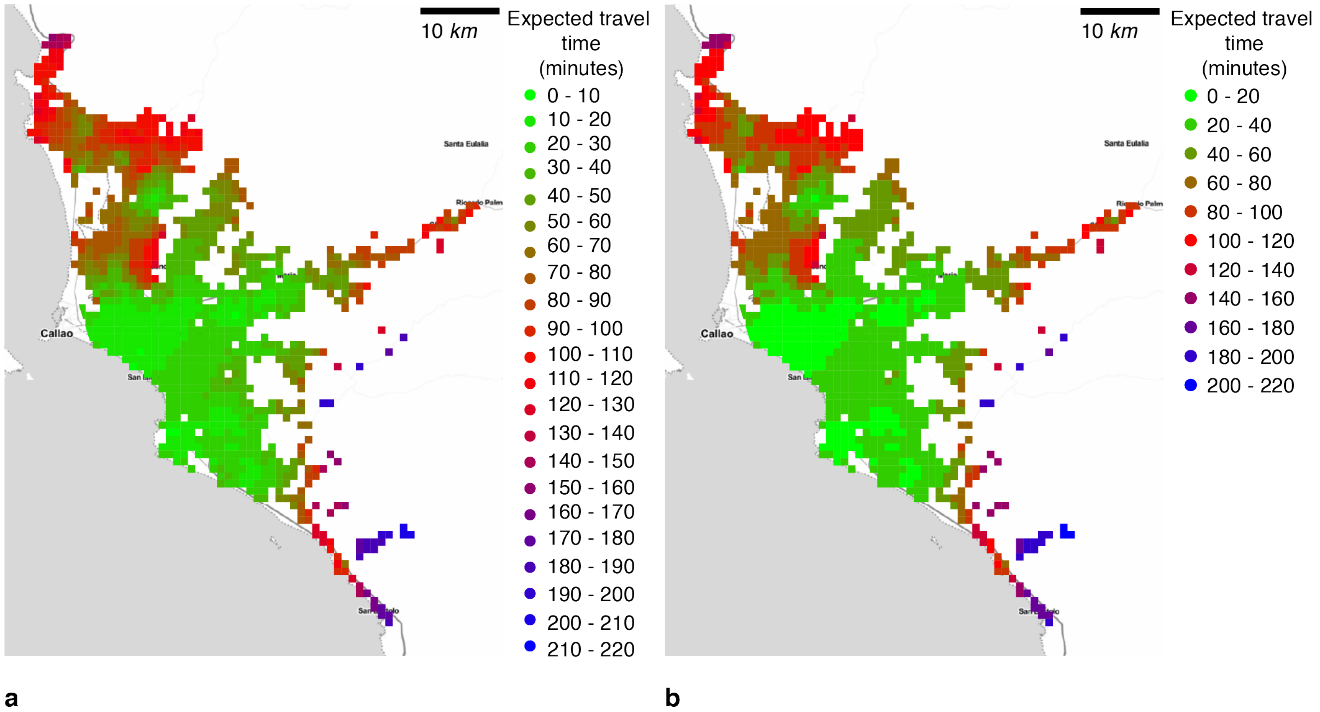

Figure S3. Compound accessibility map for Lima for the fault line scenario with a buffer of 500 with a return period of 50 and 100 years, and flood scenario of flood planning hazard map at historical return period where **(a)** 10 minute bins **(b)** 20 minute bins. (a) and (b) show the accessibility detriment in the north, south, and east. Maps were created using Map Tiles by Stamen Design, CC BY 3.3 - Map data ©using Contextily 1.2.0 library for Python [maps.stamen.com].

Undisrupted		Fault lines		Floods 5 *Cm.*	
ID	Street/avenue name	ID	Street/avenue name	ID	Street/avenue name
278935579	Avenida Tupac Amaru	278935579	Avenida Tupac Amaru	278935579	Avenida Tupac Amaru
336853460	Avenida Tupac Amaru	320750256	Avenida 9 de Diciembre	320750256	Avenida 9 de Diciembre
604415502	Avenida Tupac Amaru	320750256	Avenida 9 de Diciembre	320750256	Avenida 9 de Diciembre
295315809	Avenida Tupac Amaru	761576532	Avenida 9 de Diciembre	761576532	Avenida 9 de Diciembre
320750256	Avenida 9 de Diciembre	336853460	Avenida Tupac Amaru	359190907	Avenida Almirante Miguel Grau
320750256	Avenida 9 de Diciembre	604415502	Avenida Tupac Amaru	279400979	Via Expresa Almirante Miguel Grau
761576532	Avenida 9 de Diciembre	295315809	Avenida Tupac Amaru	320757334	Avenida Almirante Miguel Grau
670372972	Avenida Cesar Vallejo	670372972	Avenida Cesar Vallejo	284929200	Avenida Proceres de la Independencia
359190907	Avenida Almirante Miguel Grau	279400979	Via Expresa Almirante Miguel Grau	344364182	Avenida Proceres de la Independencia
279400979	Via Expresa Almirante Miguel Grau	359190907	Avenida Almirante Miguel Grau	359190907	Avenida Almirante Miguel Grau

Table S3. Top ten streets with highest criticality for the undisrupted (baseline), fault lines with a return period of 50 and 100 years, and 5 *cm* flood depth scenarios of flood planning hazard map at historical return period for Lima.

Undisrupted		Max		Sum	
ID	Street/avenue name	ID	Street/avenue name	ID	Street/avenue name
278935579	Avenida Tupac Amaru	278935579	Avenida Túpac Amaru	278935579	Avenida Tupac Amaru
320750256	Avenida Tupac Amaru	320750256	Avenida 9 de Diciembre	320750256	Avenida 9 de Diciembre
320750256	Avenida Tupac Amaru	320750256	Avenida 9 de Diciembre	320750256	Avenida 9 de Diciembre
761576532	Avenida Tupac Amaru	761576532	Avenida 9 de Diciembre	761576532	Avenida 9 de Diciembre
359190907	Avenida 9 de Diciembre	359190907	Avenida Almirante Miguel Grau	359190907	Avenida Almirante Miguel Grau
279400979	Avenida 9 de Diciembre	279400979	Vía Expresa Almirante Miguel Grau	279400979	Via Expresa Almirante Miguel Grau
320757334	Avenida 9 de Diciembre	320757334	Avenida Almirante Miguel Grau	320757334	Avenida Almirante Miguel Grau
670372972	Avenida Cesar Vallejo	284929200	Avenida Próceres de la Independencia	670372972	Avenida Cesar Vallejo
344364182	Avenida Almirante Miguel Grau	344364182	Avenida Próceres de la Independencia	344364182	Avenida Proceres de la Independencia
284929200	Via Expresa Almirante Miguel Grau	359190907	Avenida Almirante Miguel Grau	284929200	Avenida Proceres de la Independencia

Table S4. Top ten streets with the highest criticality for the undisrupted (baseline), compound max, and compound sum scenarios for Lima. Where compound take fault lines scenario with a return period of 50 and 100 years, and 5 *cm* flood depth scenarios of flood planning hazard map at historical return period

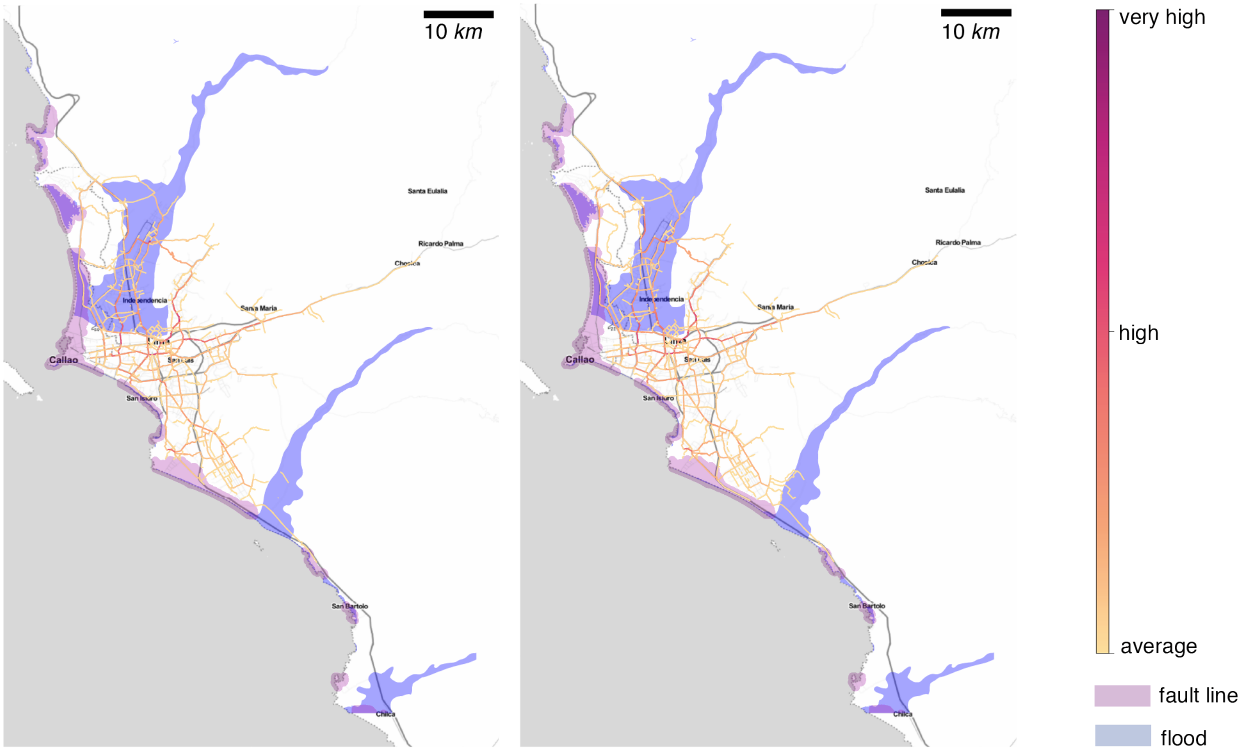

a **b**

Figure S4. Compound criticality map for Lima city where **(a)** is the compound scenario summing the criticalities from fault line scenario with a return period of 50 and 100 years and floods scenario of flood planning hazard map at historical return period, and **(b)** is the compound scenario taking the maximal value between fault line and floods.

hours	Undisrupted	Fault line	Floods 5 *cm*	Floods 15 *cm*
0 - 1	97.76%	76.40%	83.41%	74.66%
1 - 2	0.24%	16.29%	16.35%	3.04%
2 - 3	0%	0.03%	0.24%	0%
Unreachable	0%	7.28%	0%	22.30%

Table S5. Population accessibility change in undisrupted and disrupted flood with a return period of 100 years and fault lines with a return period of 500 years scenarios in Manila.

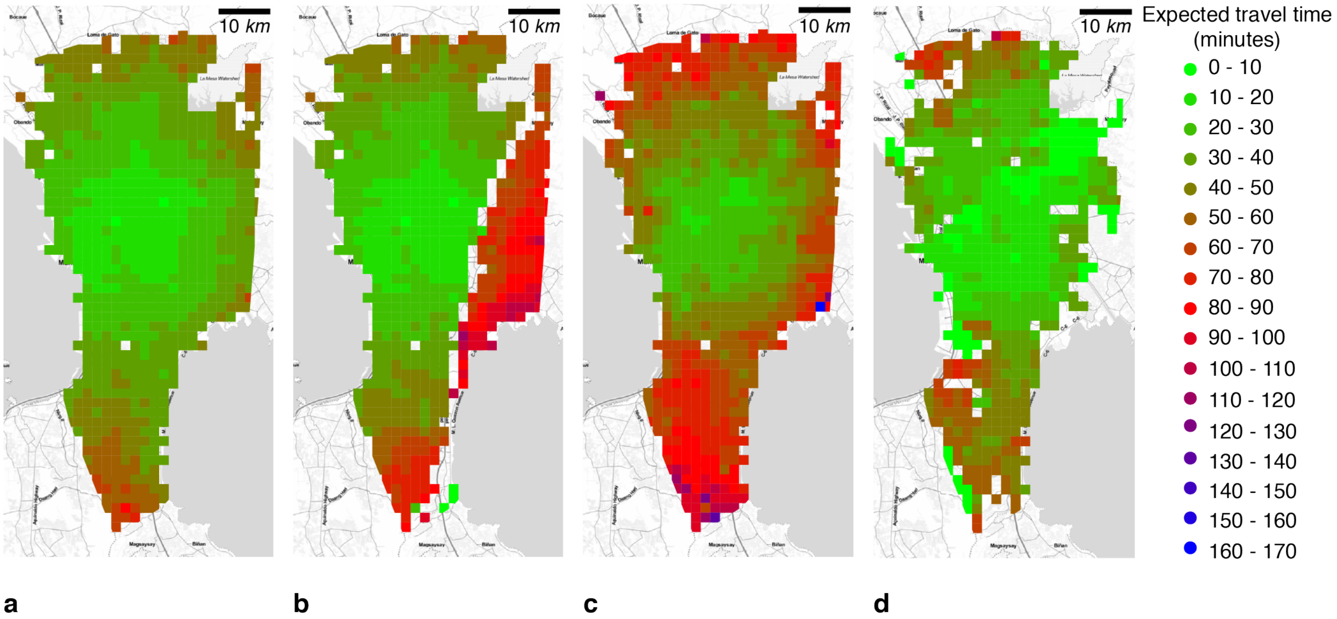

Figure S5. Accessibility map for healthcare facilities in Manila city. Where **(a)** is the undisrupted scenario, **(b)** is the fault line scenario with a buffer of 500m with a return period of 500 years, **(c)** is the flood scenario at 5 *cm*. with a return period of 100 years, and **(d)** is the flood scenario at 15 *cm* with a return period of 100 years, with a heatmap of disrupted number of trips. (b) depicts the West Valley fault downgrading the accessibility. (c) shows the decrease of accessibility time in the city's outskirts. (d) illustrates a better accessibility time due to the 22.98% of isolated population. Maps were created using Map Tiles by Stamen Design, CC BY 3.3 - Map data ©using Contextily 1.2.0 library for Python [maps.stamen.com].

Undisrupted		Fault lines		Floods 5 *Cm.*		Floods 15 *Cm.*	
ID	Street/Ave. name	ID	Street/Ave. name	ID	Street/Ave. name	ID	Street/Ave. name
354323995	EDSA	398689857	Commonwealth Ave.	846602449	East Ave.	389070663	Commonwealth Ave.
354323995	EDSA	398689855	Commonwealth Ave.	846602449	East Ave.	389070663	Commonwealth Ave.
188165109	EDSA	670619395	Commonwealth Ave.	846602449	East Ave.	389070656	Commonwealth Ave.
188165107	EDSA	670619391		936498147	East Ave.	389070663	Commonwealth Ave.
719051889	EDSA	547158016		741168401	East Ave.	910349408	Commonwealth Ave.
29255579	Pavilion 1 & 2 Road	670619390	Commonwealth Ave.	936496841	East Ave.	238942118	Commonwealth Ave.
29255579	Pavilion 1 & 2 Road	670619389	Commonwealth Ave.	234438270	East Ave.	238942118	Commonwealth Ave.
262040035		830780905	Commonwealth Ave.	234438270	East Ave.	622651354	Commonwealth Ave.
29255519		894567973		18578491	East Ave.	569325548	Commonwealth Ave.
262040033		830780905	Commonwealth Ave.	106618133	East Ave.	389070663	Commonwealth Ave.

Table S6. Top ten streets with highest criticality for the undisrupted (baseline), fault lines with a return period of 500 years, 5 *cm* flood depth with a return period of 100 years, and 15 *cm* flood depth with a return period of 100 years scenarios.

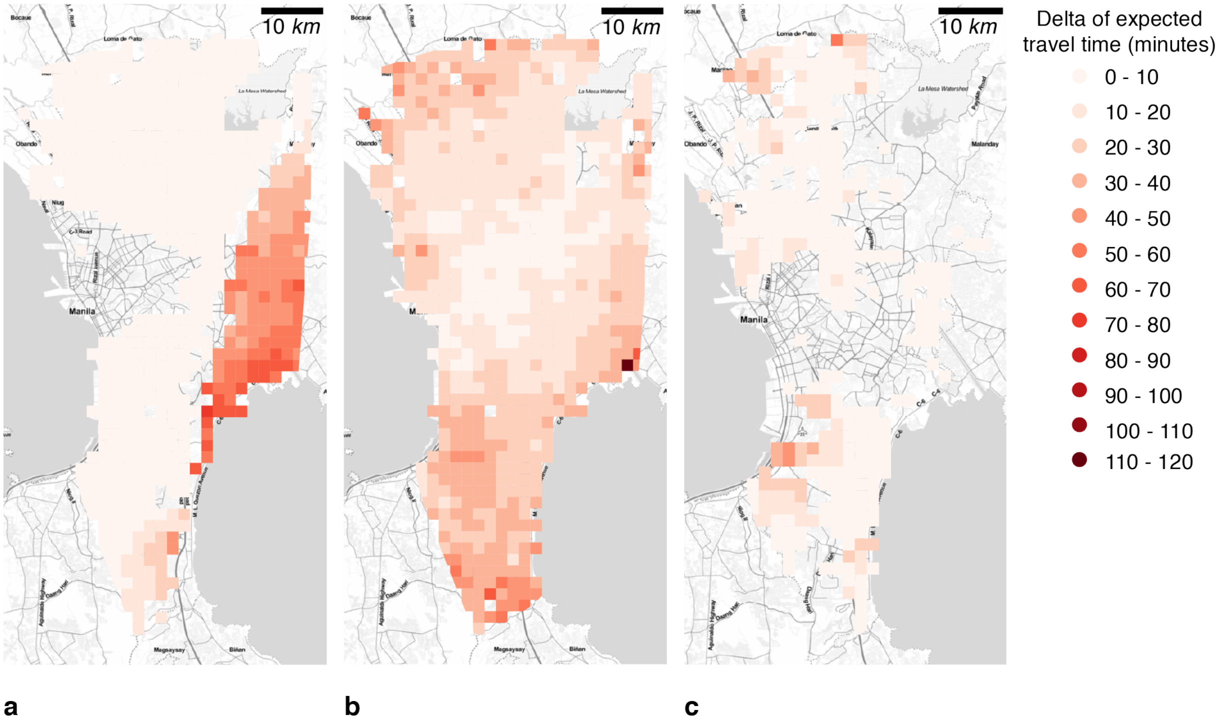

Figure S6. Time accessibility map difference for healthcare facilities in Manila city undisrupted scenario compared to **(a)** the fault line scenario with a buffer of 500 m with a return period of 500 years, **(b)** the flood scenario with at 5 *cm*. with a return period of 100 years, and **(c)** the flood scenario with at 15 *cm*. with a return period of 100 years. (b) shows the decrease of accessibility time in the city's outskirts. (c) illustrates a better accessibility time due to the 22.30% of isolated population. Maps were created using Map Tiles by Stamen Design, CC BY 3.3 - Map data ©using Contextily 1.2.0 library for Python [maps.stamen.com].

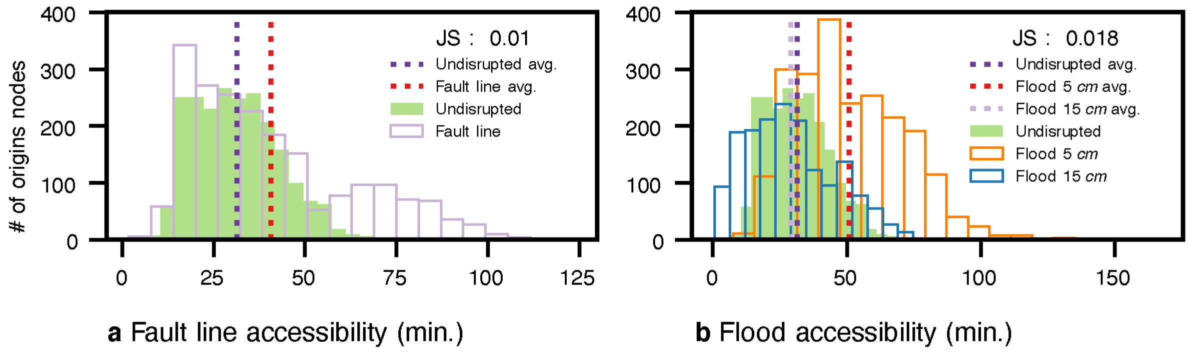

Figure S7. Distribution of accessibility time for healthcare facilities in Manila city. Where **(a)** is the fault line scenario with a buffer of 500 m with a return period of 500 years, **(b)** is the flood scenario at 5 *cm* and 15 *cm* with a return period of 100 years. The Jensen-Shannon divergence are 0.01, 0.002, and 0.018 for fault line, flood at 5 *cm* and flood at 5 *cm* with respect to undisrupted scenario.

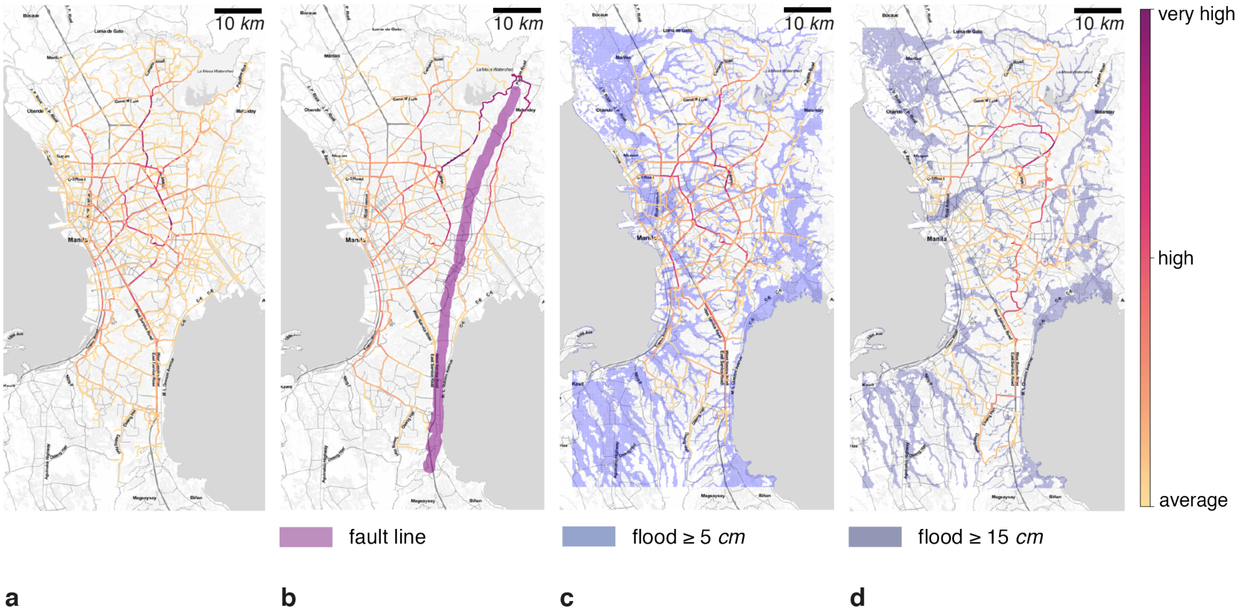

Figure S8. Criticality map for Manila road network infrastructure. Where **(a)** is the criticality in the undisrupted scenario, **(b)** is the criticality in the fault line scenario with a buffer of 500 m with a return period of 500 years, **(c)** is the is the criticality at 5 *cm* flood scenario with a return period of 500 years, and **(d)** is the is the criticality at 15 *cm* flood scenario with a return period of 100 years. (b) illustrates the criticality increment over the West Valley fault. (c) shows a more homogeneous criticality increment over the Metro Manila center. (d) depicts less criticality due to the 22.30% of isolated population. Maps were created using Map Tiles by Stamen Design, CC BY 3.3 - Map data ©using Contextily 1.2.0 library for Python [maps.stamen.com].

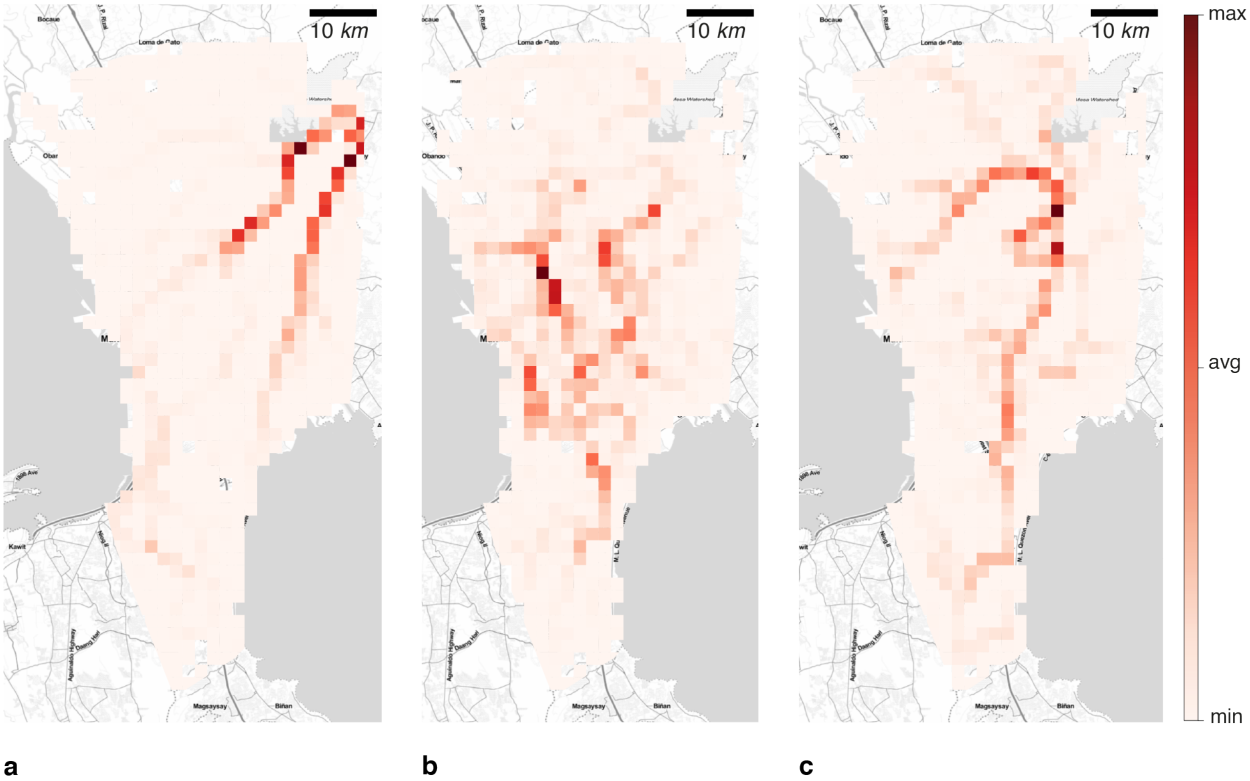

Figure S9. Criticality variation map between undisrupted and disrupted scenarios for healthcare facilities in Manila city. Where **(a)** is the fault line scenario with a buffer of 500 meters with a return period of 500 years, **(b)** is the flood scenario with at 5 *cm*. with a return period of 100 years, and **(c)** is the flood scenario with at 15 *cm* with a return period of 100 years. (a) illustrates the criticality increment over the West Valley fault. (b) shows a more homogeneous criticality increment over the Metro Manila center. (c) depicts less criticality due to the 22.30% of isolated population. Maps were created using Map Tiles by Stamen Design, CC BY 3.3 - Map data ©using Contextily 1.2.0 library for Python [maps.stamen.com].

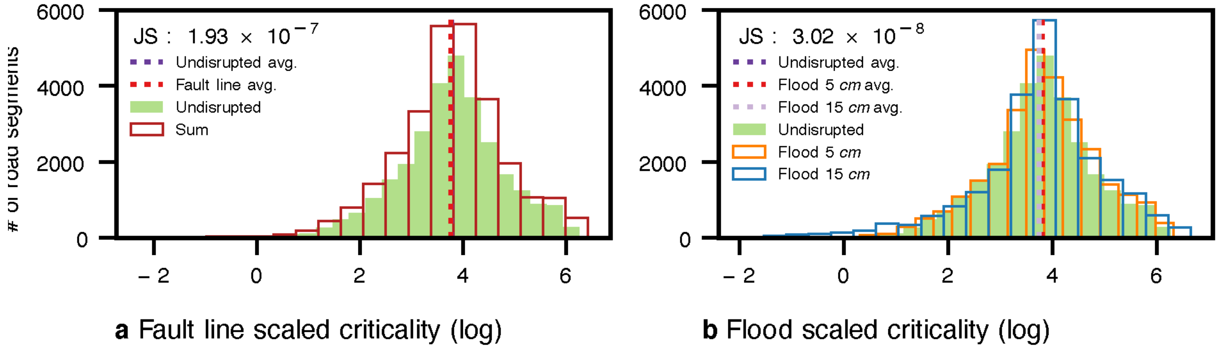

Figure S10. Criticality distribution for Manila city in log scale. Where **(a)** is the fault line scenario with a return period of 500 years, **(b)** is the flood scenario at 5 *cm*, and 15 *cm* with a return period of 100 years. The Jensen-Shannon divergence are 1.93×10^{-7}, 3.02×10^{-8}, and 2.42×10^{-7} for fault line (a), flood at 5 *cm*, and flood at 5 *cm* (b) with respect to the undisrupted scenario.

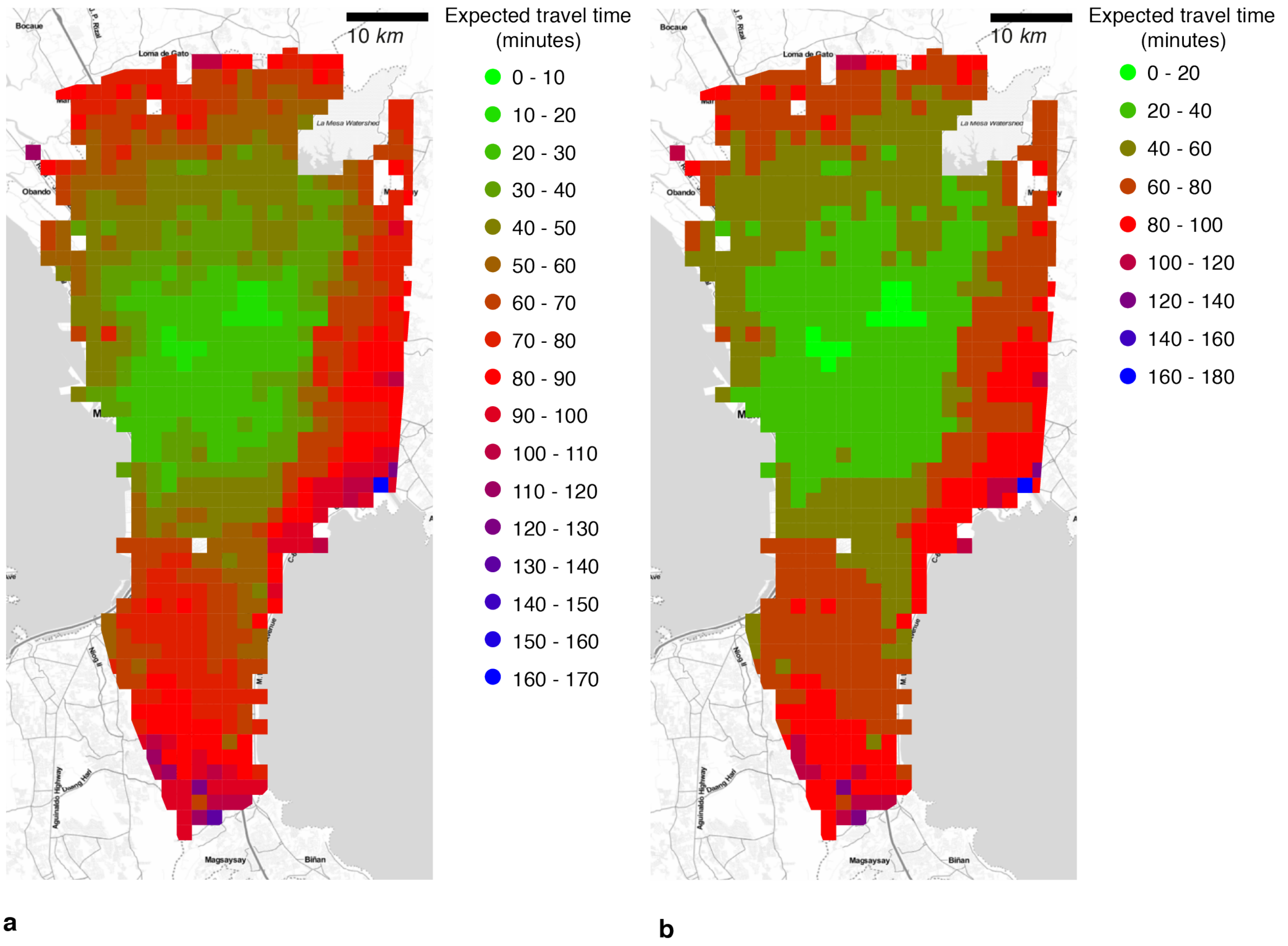

Figure S11. Compound accessibility map with hazard fault line with a return period of 500 years and flood with a return period of 100 years for Manila city where **(a)** 10 minute bins **(b)** 20 minute bins. (a) and (b) shows a small accessibility time in the Metro Manila center, which declines as one moves away from the city center. Maps were created using Map Tiles by Stamen Design, CC BY 3.3 - Map data ©using Contextily 1.2.0 library for Python [maps.stamen.com].

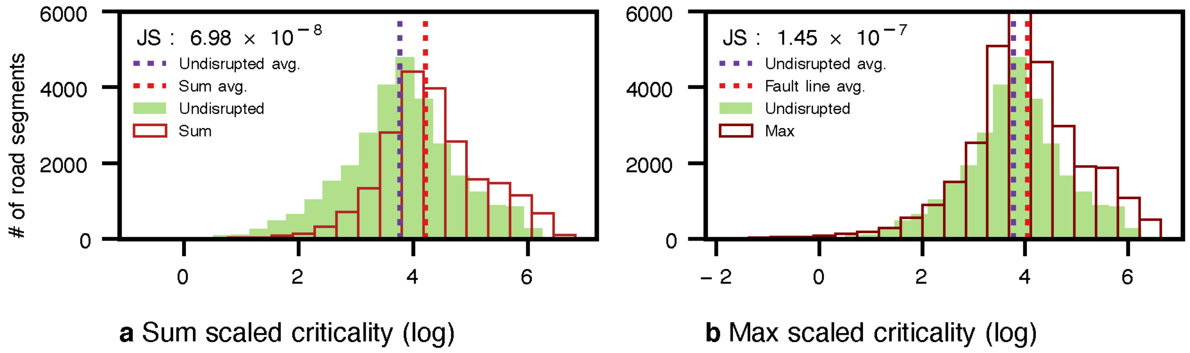

Figure S12. Criticality distribution for compound hazard fault line with a return period of 500 years and flood with a return period of 100 years for Manila city in log scale. Where **(a)** is the compound hazard flood and fault line taking the sum of both values, and **(b)** is the compound hazard flood and fault line taking the maximal value. The Jensen-Shannon divergence are 6.98×10^{-8} and 1.45×10^{-7} for (a) and (b) with respect to the undisrupted scenario. Maps were created using Map Tiles by Stamen Design, CC BY 3.3 - Map data ©using Contextily 1.2.0 library for Python [maps.stamen.com].

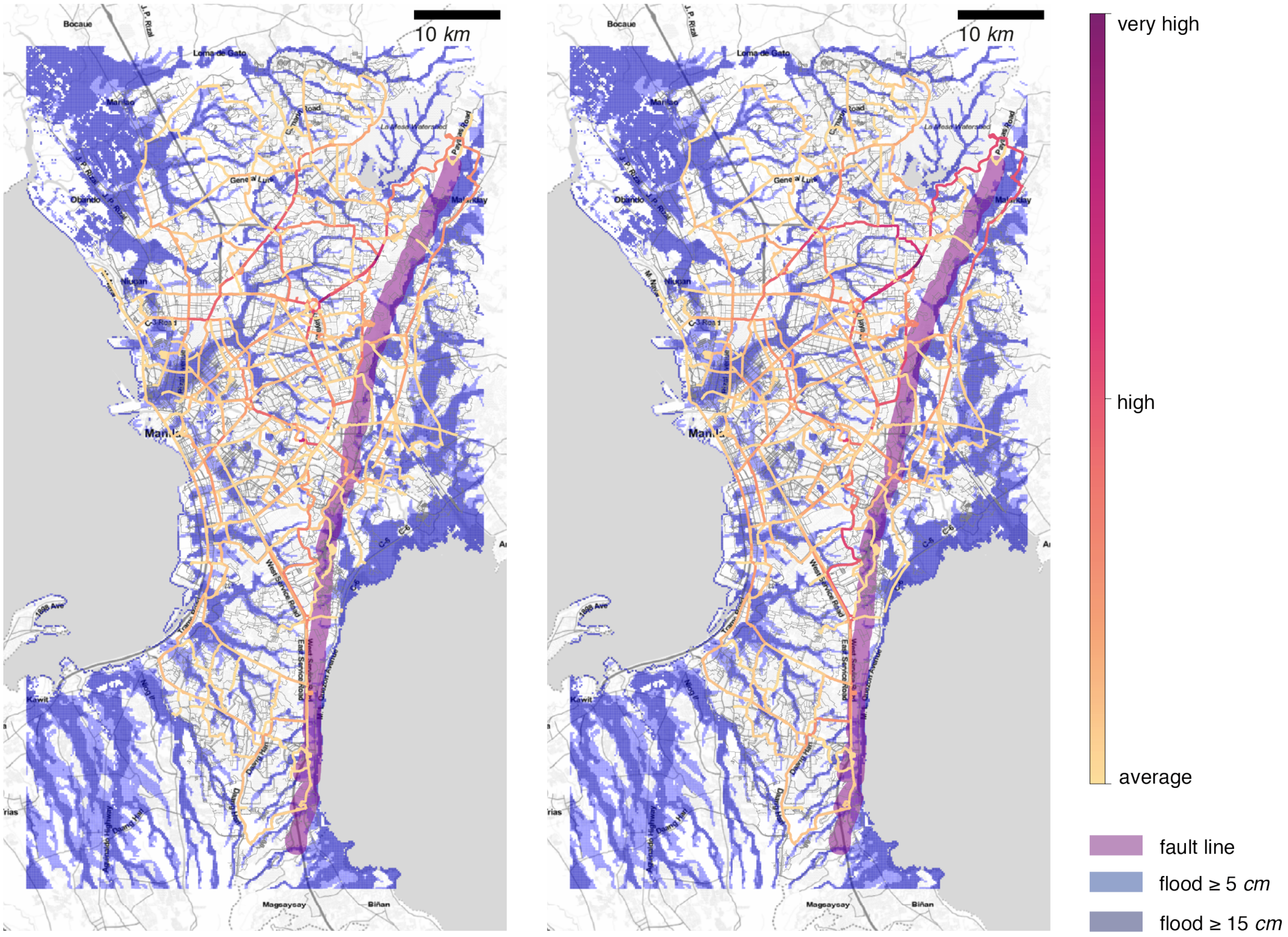

a **b**

Figure S13. Criticality distribution for compound hazard fault line with a return period of 500 years and flood with a return period of 100 years for Manila city. Where **(a)** is the compound hazard flood and fault line taking the sum of both values, and **(b)** is the compound hazard flood and fault line taking the maximal value. The Jensen-Shannon divergence are 1.66×10^{-8} and 1.46×10^{-8} for (a) and (b) with respect to undisrupted scenario.
